# *Entamoeba histolytica* EhCP112 Dislocates and Degrades Claudin-1 and Claudin-2 at Tight Junctions of the Intestinal Epithelium

**DOI:** 10.3389/fcimb.2017.00372

**Published:** 2017-08-16

**Authors:** Patricia Cuellar, Elizabeth Hernández-Nava, Guillermina García-Rivera, Bibiana Chávez-Munguía, Michael Schnoor, Abigail Betanzos, Esther Orozco

**Affiliations:** ^1^Departamento de Infectómica y Patogénesis Molecular, Centro de Investigación y de Estudios Avanzados del Instituto Politécnico Nacional Mexico, Mexico; ^2^Departamento de Biomedicina Molecular, Centro de Investigación y de Estudios Avanzados del Instituto Politécnico Nacional Mexico, Mexico; ^3^Consejo Nacional de Ciencia y Tecnología Mexico, Mexico

**Keywords:** *Entamoeba histolytica*, tight junctions, EhCPADH complex, EhCP112, Caco-2 cells, murine amoebiasis model, claudins, paracellular pathway

## Abstract

During intestinal invasion, *Entamoeba histolytica* opens tight junctions (TJs) reflected by transepithelial electrical resistance (TEER) dropping. To explore the molecular mechanisms underlying this, we studied *in vitro* and *in vivo* the damage produced by the recombinant *E. histolytica* cysteine protease (rEhCP112) on TJ functions and proteins. rEhCP112 reduced TEER in Caco-2 cells in a dose- and time-dependent manner; and EhCP112-overexpressing trophozoites provoked major epithelial injury compared to control trophozoites. rEhCP112 penetrated through the intercellular space, and consequently the ion flux increased and the TJs fence function was disturbed. However, macromolecular flux was not altered. Functional *in vitro* assays revealed specific association of rEhCP112 with claudin-1 and claudin-2, that are both involved in regulating ion flux and fence function. Of note, rEhCP112 did not interact with occludin that is responsible for regulating macromolecular flux. Moreover, rEhCP112 degraded and delocalized claudin-1, thus affecting interepithelial adhesion. Concomitantly, expression of the leaky claudin-2 at TJ, first increased and then it was degraded. *In vivo*, rEhCP112 increased intestinal epithelial permeability in the mouse colon, likely due to apical erosion and claudin-1 and claudin-2 degradation. In conclusion, we provide evidence that EhCP112 causes epithelial dysfunction by specifically altering claudins at TJ. Thus, EhCP112 could be a potential target for therapeutic approaches against amoebiasis.

## Introduction

*Entamoeba histolytica* is the causative agent of amoebiasis, responsible for up to 100,000 deaths worldwide per year (Mortimer and Chadee, 2010). Trophozoites colonize the large intestine producing watery and bloody diarrhea (Espinosa-Cantellano and Martínez-Palomo, 2000; Haque et al., 2003). The intestinal epithelium forms a barrier that prevents pathogens entrance and regulates nutrient acquisition (Hodges and Gill, 2010). This barrier is constituted by a highly-organized monolayer of polarized epithelial cells that are bound mainly through tight junctions (TJs), adherens junctions (AJs), and desmosomes (DSMs) (Sousa et al., 2005). TJs are localized at the most apical region of the intercellular space and are the first barrier for pathogens (Sousa et al., 2005). TJs are formed by transmembrane proteins such as occludin and claudins, and cytoplasmatic plaque proteins such as ZO-1 and ZO-2, which bind to the actin cytoskeleton (Liang and Weber, 2014). Occludin participates in the regulation of the macromolecules flux, while claudins mediate ion flux control. Such paracellular flux is considered as a TJ gate function. Moreover, claudins also restrict proteins and lipids diffusion within membranes, thus contributing to epithelial polarization. This function is known as TJs fence function (Lingaraju et al., 2015).

During intestinal invasion and colonization, pathogens destabilize TJs by different mechanisms. In rotavirus, VP8 protein alters the localization of occludin and ZO-1 (Nava et al., 2004). *Clostridium perfringens* secretes an enterotoxin which binds to and disintegrates claudins (Sonoda et al., 1999); whereas, enteropathogenic *Escherichia coli* activates the epithelial RhoA kinase, contracting actin perijunctional ring and opening TJs (Matsuzawa et al., 2005); *Salmonella* sp. activate the epidermal growth factor receptor pathway and elevate the expression of claudin-2 in the colon (Zhang et al., 2013). The protozoan *Giardia duodenalis* affects the distribution of claudin-1 and ZO-1 and decreases intestinal transepithelial electrical resistance (TEER) (Maia-Brigagao et al., 2012).

*E. histolytica* trophozoites damage the intestinal epithelium and drop TEER in cultured epithelial cells, such as MDCK (Martinez-Palomo et al., 1985; Betanzos et al., 2013), T84 (Leroy et al., 2000; Lejeune et al., 2011), and Caco-2 (Li et al., 1994) cell monolayers. Prostaglandin E_2_ (PGE_2_) (Lejeune et al., 2011) and the EhCPADH complex (Betanzos et al., 2013) drop TEER. The concerted action of these and other molecules allows the trophozoites invasion to the intestinal epithelium. *E. histolytica* has 50 putative cystein proteases (CPs), some of them are secreted and involved in the damage to epithelium (Serrano-Luna et al., 2013). Among *E. histolytica* CPs, EhCP1 cleaves key components of the host immune system, C3 complement factor, immunoglobulin G, and pro-interleukin-18 (Meléndez-López et al., 2007); EhCP2 cleaves the chemokines CCL2, CCL13, and CXCL8, and the resulting proteolysis products modulate the chemotaxis of leukocytes (Pertuz Belloso et al., 2004; Irmer et al., 2009); EhCP5 elicits the fast release of mucin by goblet cells (Cornick et al., 2016); whereas EhCP112 degrades collagen type I, gelatin, fibronectin, and hemoglobin and damages epithelial cells (Arroyo and Orozco, 1987; Garcia-Rivera et al., 1997; Banuelos et al., 2005).

EhCP112 together with EhADH (an ALIX family protein) forms the EhCPADH virulence complex. EhCP112 has a canonical catalytic domain and an RGD sequence that in other organisms interacts with integrins (Ruoslahti, 1996; Bruchhaus et al., 2003). However, at molecular level, its contribution to the epithelial damage remains unknown. It is known that pre-treatment of trophozoites with protease inhibitors or an α-EhCPADH antibody prevents injury (Betanzos et al., 2013), suggesting that EhCP112 indeed participates in TJs disruption. Here, we studied *in vitro* and *in vivo* the molecular mechanisms that EhCP112 follows to alter TJ functions and proteins. Our results showed that rEhCP112 disrupted TJs in polarized Caco-2 cells with a higher efficiency than other CPs from *E. histolytica*, increasing ion but not macromolecules flux and also affecting fence function. We also observed delocalization and degradation of claudin-1 and an increase in the claudin-2 amount. *In vivo*, rEhCP112 injured mouse intestinal epithelium, as manifested by claudin-1 and claudin-2 degradation and increased permeability.

## Materials and methods

### Cell cultures

Caco-2 (human colorectal adenocarcinoma) from the C2BBe1 lineage and MDCK (Madin Darby canine kidney) type I (Cereijido et al., 1978; Sambuy et al., 2005) cells (passages: 15–37 and 20–100, respectively) were grown in DMEM medium (Gibco) supplemented with penicillin (100 IU/ml), streptomycin (100 mg/ml) (*in vitro*), 10% fetal bovine serum (Gibco), and insulin (0.08 U/ml) (Eli Lilly), at 37°C in a 5% CO_2_ atmosphere (Natoli et al., 2012). Cell confluence was verified by TEER measurement and by phase contrast microscopy. *E. histolytica* trophozoites, strain HM1:IMSS, clone A (Orozco et al., 1983) were axenically cultured at 37°C in TYI-S-33 medium and harvested during logarithmic growth phase (Diamond et al., 1978). All experiments presented here were performed at least three independent times by duplicate.

### Production of recombinants EhCPs

To produce and activate rEhCP1, rEhCP2, rEhCP5, and rEhCP112 recombinant proteases, *E. coli* BL21 DE3 bacteria were transformed with the pQE80L-*ppEhcp112* or pet15-*ppEhcpa1* or pet15-*ppEhcpa2* or pet15-*ppEhcpa5* constructions (kindly donated by Dr. Jaime Ortega from the Department of Biotechnology and Bioengineering, CINVESTAV-IPN, México). Recombinant proteins were induced with 1 mM IPTG and recovered from the inclusion bodies, using the solubilization buffer (20 mM sodium phosphate, pH 7.4, 0.5 M NaCl, 10 mM imidazole, and 8 M urea). Recombinant enzymes were purified using a HisPur cobalt resin (Thermo Fisher Scientific) and refolded in a PD-10 desalting column (GE Healthcare Science) with the refolding buffer (5 mM CaCl_2_, 0.02% SDS, and 100 mM Tris–HCl pH 8.0). Recombinant proteins were activated with 0.05% β-mercaptoethanol (β-ME), during 20 min at 25°C. The proteolytic activity of the enzymes was confirmed by zymogram assays in 12% polyacrylamide gels (PAGE) co-polymerized with 0.1% gelatin and stained with Coomassie blue solution. We also used the Z-Phe-Arg substrate degradation and releasing of 7-amino-4-methylcoumarin (AMC) was measured in a fluorimeter (Fluoroskan Ascent FL) by excitation and emission wavelengths at 355 and 460 nm, respectively. Bovine serum albumin (BSA) was used as a negative control.

### Antibodies

For EhCP112 immunodetection, rabbit polyclonal antibodies against a specific EhCP112 C-terminus peptide (N-431KYHSNSTYVQFYNHT444-C) were used (Betanzos et al., 2013). For immunoprecipitation assays, a polyclonal antibody against EhCP112 (α-EhCP112) was obtained after three immunizations (each 2 weeks) of New Zealand male rabbits with 300 μg of rEhCP112p diluted in TiterMax® (Sigma) adjuvant. Before immunization, rabbits were bled to obtain pre-immune serum (PS). Other primary antibodies used were: mouse monoclonal α-actin (kindly donated by Dr. Jose Manuel Hernández from the Department of Cellular Biology, CINVESTAV-IPN, México), mouse monoclonal α-claudin-1 (Invitrogen, cat number: 51-9000), α-claudin-2, α-claudin-4 (Thermo Scientific, cat number: 32-5600 and 32-9400, respectively), α-occludin (Invitrogen, cat number: 33-1500), α-mucin-2 (Thermo Scientific, cat number: MA1-35701) and α-GAPDH (Santa Cruz, cat number: sc-365062) antibodies; and rabbit polyclonal α-ZO-2, α-ZO-1 (Invitrogen, cat number: 37-4700 and 40-2200, respectively) anti-α/β tubulin (α-tubulin) (Cell Signaling, cat number: 2148S) antibodies. Secondary antibodies derived from goat included: α-rabbit and α-mouse HRP-labeled IgGs (1:10,000) (Life technologies G21234 and 62–6,520, respectively); and α-rabbit and α-mouse FITC and TRITC-labeled IgGs (1:150) (Zymed, 65–6,111 and Life technologies F-2761, respectively).

### Incubation of epithelial cells with rEhCP112a

rEhCP112a (10–30 μg/cm^2^) or trophozoites (10^5^/cm^2^; 2:1 cells to trophozoites ratio) or 5 mM EDTA or refolding buffer were added to the apical side of confluent Caco-2 or MDCK cells and incubated for different times at 37°C.

### rEhCP112p labeling and interaction with Caco-2 cells

rEhCP112p was labeled with Alexa 647 fluorescent dye, following the manufacturer recommendations (Molecular Probes). Briefly, 100 μg of rEhCP112p were equilibrated with 0.1 M sodium bicarbonate to allow the adequate succinimidyl ester reaction with primary amines of the protein, to form stable dye-protein conjugates. Confluent Caco-2 cells were grown on coverslips and Alexa-labeled rEhCP112p was added for 0, 0.5, 1, and 5 min to the apical side of the cells. After washing five times with PBS, pH 6.8, cells were fixed with absolute ethanol for 30 min at −20°C and nuclei were stained for 5 min with 2.5 μg/ml of 4′,6-diamidino-2-phenylindole (DAPI) (Zymed). Cells were mounted using Vectashield (Vector laboratories) and analyzed in a confocal microscope (Leica TCS_SP5_MO) through *Z*-stack sections of 0.5 μm and *xy*- and *zy*-planes. In all cases, 10 fields were analyzed per condition. Representative images were selected for each time.

### TEER assays

Different concentrations of rEhCP112a (0–30 μg/cm^2^) were added to the apical side of confluent Caco-2 or MDCK cells grown on transwell filters (6.5 mm of diameter and 0.4 μm pore) (Corning) and then, TEER was measured using an EVOM epithelial voltmeter (World Precision Instruments) during 90 min. In some assays, rEhCP112a was pre-incubated for 10 min with 20 μg/ml of E-64 or 10 μg of α-EhCP112 antibody, before the enzyme was added to Caco-2 cells. rEhCPA1, rEhCP2 or rEhCP5 (10 μg/cm^2^) or trophozoites (10^5^/cm^2^; 2:1 ratio) or papain were added to Caco-2 cells. Each transwell measurement was normalized accordingly to its initial value (above 1,000 Ω/cm^2^) before treatment (Betanzos et al., 2013). During the TEER assays, cells were maintained at 37°C to avoid temperature changes. To verify the plasma membrane integrity, treated cells were stained by 2 μg/ml of propidium iodide (PI) for 10 min. We also verified cell viability using the Sytox green reagent (Thermo Scientific), following the manufacturer instructions. Cells were washed and observed through a laser confocal microscope.

### Transfection of trophozoites

*Ehcp112* complete gene was PCR amplified with specific primers containing *Bam*HI and *Kpn*I restriction sites (forward: 5′-GGGGTACCATGACAGCGATTGTTGTCGCTTTTTT-3′, reverse: 5′-CGGGATCCTTACTTATCGTTCGTCATCCTTGTAATCGATTGTATGATTGTAGAATTGG-3′) under the following conditions: 95°C 15 s, 60°C 30 s, 72°C 90 s during 30 cycles. Then, the PCR product was cloned into pJET1.2 plasmid (Fermentas) according to the manufacturer recommendations. The *Ehcp112*/pJET construction was digested and the *Ehcp112* fragment was subcloned in the *pExEhNeo* plasmid (Hamann et al., 1995). This plasmid was amplified in *E. coli* DH5α, purified by Qiagen Midi Kit (Qiagen) and automatically sequenced. Transfection was performed as described (Avalos-Padilla et al., 2015). Transfected trophozoites were selected with increasing concentrations of G-418 (a neomycin analog) until stable growth was achieved (20 μg/ml). Overexpression of the *Ehcp112* gene was verified by RT-PCR by specific primers and using *s2* ribosomal gene as internal control. WB assays were performed using α-EhCP112 (1:5,000) and α-actin (1:3,000) antibodies. Laser confocal microscopy assays were performed using α-EhCP112 (1:100) antibodies in paraformaldehyde fixed and triton X-100 permeabilized trophozoites and nuclei were stained by propidium iodide.

### Paracellular permeability of epithelial cell lines

Paracellular permeability of Caco-2 and MDCK cells was determined by red ruthenium dye (RR) penetration and by FITC-dextran diffusion assays. For RR penetration assays, cell monolayers were incubated for 30 min with rEhCP112a (10 μg/cm^2^) or with trophozoites or EDTA (5 mM) or refolding buffer. Then, cells were fixed with 2.5% glutaraldehyde and incubated for 1 h at room temperature (RT) with 1% osmium tetraoxide and 5 μg/ml of RR. Samples were dehydrated by incubation in increasing concentrations of ethanol and propylene oxide and embedded in Polybed resin (Polysciences). Ultrathin sections (60 nm) were obtained and analyzed through a Jeol 1011 transmission electron microscope.

For FITC-dextran assays, 3 mg/ml of FITC-dextran (2–4 kDa) (Sigma Aldrich) were added to the apical side of confluent epithelial cells. Then, rEhCP112a (10 μg/cm^2^) or trophozoites or EDTA or refolding buffer were added to the upper chamber of transwells and incubated for 90 min at 37°C with gentle shaking. Samples from the basal chamber were collected each 30 min and the diffused fluorescent tracer was measured in the fluorimeter by excitation and emission wavelengths at 492 and 520 nm, respectively. Emission values were converted to FITC-dextran concentration, using a standard curve (Matter and Balda, 2003).

### Bodipy diffusion assays

BSA-BODIPY FL C_12_-Sphingomyelin (Molecular Probes) (50 μg) re-suspended in P buffer (10 mM HEPES pH 7.4, 145 mM NaCl, 1 mM sodium pyruvate, 10 mM glucose, 3 mM CaCl_2_, and 3.4 mg of ultrapure BSA) was added to confluent Caco-2 cell monolayers grown on coverslips and then, incubated for 10 min at 4°C. After washing three times with P buffer, rEhCP112a (10 μg/cm^2^) or refolding buffer or EDTA were added to the apical side of the cells. Samples were processed for immunofluorescence confocal microscopy, using the α-occludin antibody as a TJs marker, as described below. Images were acquired in the *zy*-plane, to visualize Bodipy diffusion (Matter and Balda, 2003).

### Cellular lysis and WB assays

Caco-2 cells were incubated for 2, 10, and 30 min at 37°C with 10 μg/cm^2^ of rEhCP112a or E-64 (20 μg/ml for 10 min) pre-treated rEhCP112a or live trophozoites. After washing twice with PBS, cells were scrapped with a rubber policeman and lysed in RIPA buffer [40 mM Tris-HCl pH 7.6, 150 mM NaCl, 2 mM EDTA, 10% glycerol, 1% Triton X-100, 0.5% sodium deoxycholate, 0.2% SDS, 1 mM PMSF and 1 mM Complete™ protease inhibitor cocktail (Roche)] under continuous and vigorous shaking. Extracts were sonicated three times for 5 s, centrifuged for 15 min at 15,300 × g to eliminate cellular debris and boiled with sample buffer (50 mM Tris-HCl pH 6.8, 2% SDS, 10% glycerol, 1% β-mercaptoethanol, 12.5 mM EDTA, and 0.02 % bromophenol blue; Betanzos et al., 2013). Protein samples were separated by 6, 8, 10, or 15% sodium dodecyl sulfate (SDS)-PAGE, transferred to nitrocellulose membranes and incubated 1 h with 5% non-fat milk. Membranes were incubated overnight (ON) with α-EhCP112 (1:5,000) or α-occludin (1:1,000) or α-claudin-1 (1:1,500) or α-claudin-2 (1:500) or α-claudin-4 (1:500) or α-ZO-1 (1:500) or α-ZO-2 (1:400) or α-Tub (1:3,000) antibodies. After washing, membranes were incubated for 1 h with the corresponding HRP-labeled secondary antibodies (1:10,000). Protein bands were visualized by chemiluminescence using the Pierce ECL-Plus kit (Thermo Fisher) and the MicroChemi system (Biostep). Densitometry analyses were performed using tubulin as loading control and employing the ImageJ software.

### Immunofluorescence assays

Confluent Caco-2 cells grown on coverslips were incubated with 10 μg/cm^2^ of rEhCP112a during 2–30 min. After washing five times with PBS pH 6.8, cells were fixed with absolute ethanol for 30 min at −20°C, blocked with 0.5% BSA and incubated ON with α-EhCP112 (1:150) and α-claudin-1 (1:50) or α-claudin-2 (1:40) or α-occludin (1:100) or α-ZO-1 (1:50) antibodies. Cells were washed and incubated with specie-specific FITC- or TRITC-labeled antibodies (1:150) (Zymed). DAPI was added to stain nuclei and cells were mounted and analyzed by confocal microscopy as above.

### Immunoprecipitation assays

Caco-2 cell monolayers were incubated with 10 μg/cm^2^ of rEhCP112p for 10 min at 37°C and then, cells were lysed with RIPA/HO buffer (1:1 vol/vol), HO buffer was composed of 50 mM HEPES pH 7.5, 150 mM NaCl, 1 mM EGTA, 1.5 mM MgCl_2_, 10% glycerol, 1% Triton X-100, and 1 mM Complete™ protease inhibitor cocktail and incubation was carried out for 20 min at 4°C. Then, lysates were incubated for 3 h with Protein G-Sepharose (Invitrogen) to clear unspecific bound proteins, washed with HO buffer and centrifuged at 500 × g for 2 min. Supernatants were incubated ON at 4°C with rabbit α-EhCP112 antibody or rabbit pre-immune serum. Next, samples were mixed with rProtein G-Sepharose for 3 h, washed five times with HO buffer, boiled with sample buffer and centrifuged at 15,300 × g for 15 min (Betanzos et al., 2013). Supernatants were analyzed by SDS-PAGE and WB, using α-EhCP112 and anti-TJ proteins antibodies.

### Permeability assay *in vivo*

Evan's blue-based *in vivo* colon permeability assays were performed as described (Lange et al., 1994; Citalán-Madrid et al., 2017). Pathogen-free C57/BL6 male mice (6–8 weeks old, ~25 g each, *n* = 6) were intraperitoneally anesthetized with 125 mg/kg of ketamine hydrochloride (Sanofi) and 12.5 mg/kg of xylazine (Phoenix Scientific) and then, rectally inoculated with rEhCP112a (50 μg in 300 μl of refolding buffer) or trophozoites (10^6^ cells in 300 μl) or with refolding buffer alone (300 μl) for 30 min. After laparotomy, a 22G polyethylene tube was inserted into the colon adjacent to the cecum and ligated. Then, remaining stool was rinsed out, and 1 ml of 1.5% Evan's blue dye (Sigma Aldrich) was instilled for 15 min. After washing with PBS until the perianal washout was clear, animals were euthanized and the colon extracted. The colon was longitudinally opened and rinsed again with PBS, followed by 1 ml of 6 mM N-acetylcysteine to remove dye within the mucus. Colons were incubated in 2 ml of formamide ON at RT and the extracted dye was spectrophotometrically measured at 610 nm. Values were expressed as arbitrary units per gram of tissue.

### Colon epithelium extraction

Colon of mice treated as above were scraped in the luminal side (Perreault and Beaulieu, 1996) and the epithelium layer was re-suspended in RIPA buffer with protease inhibitors (1 mM PMSF and 1 mM Complete™ protease inhibitor cocktail) for 30 min under continuous and vigorous shaking at 4°C. Tissue extracts were sonicated three times for 30 s, centrifuged for 15 min at 15,300 × g to eliminate undissolved cellular debris and analyzed by WB assays.

### Immunofluorescence assays of mice colon

Mouse colon samples were placed in tissue freezing medium (Leica Biosystems) and snap-frozen in liquid nitrogen for 5 min. Frozen tissue sections (10 μm) were mounted in gelatin embedded coverslips, frozen at −70°C during 1 week, fixed with absolute ethanol for 30 min at −20°C and processed for immunofluorescence staining as above.

### Statistical analysis

All data shown are representative of three independent experiments performed by duplicate unless stated otherwise. GraphPad Prism 5 software was used for statistical analysis. Data were analyzed by two tailed Student *t*-test. Statistical significance was assumed when ^*^p < 0.05, ^**^p < 0.01, or ^***^p < 0.001. All results are displayed as mean with standard error.

### Ethics statement

The Centre for Research and Advanced Studies (CINVESTAV) fulfills the standard of the Mexican Official Norm (NOM-062-ZOO-1999) “Technical Specifications for the Care and Use of Laboratory Animals” based on the Guide for the Care and Use of Laboratory Animals “The Guide,” 2011, NRC, USA with the Federal Register Number BOO.02.03.02.01.908, awarded by the National Health Service, Food Safety and Quality (SENASICA) belong to the Animal Health Office of the Secretary of Agriculture, Livestock, Rural Development, Fisheries and Food (SAGARPA), an organization that verifies the state compliance of such NOM in Mexico. The Institutional Animal Care and Use Committee (IACUC/ethics committee) from CINVESTAV as the regulatory office for the approval of research protocols, involving the use of laboratory animals and in fulfillment of the Mexican Official Norm, has reviewed and approved all animal experiments (Protocol Number 0505-12, CICUAL 001).

## Results

### rEhCP112a causes a drop in TEER in a concentration and time-dependent manner

*E. histolytica* is a phagocytic and cytolytic parasite in which several molecules contribute to its pathogenicity (Thibeaux et al., 2013). The EhCPs family is composed by 50 members in the genome (Que and Reed, 2000) (http://amoebadb.org) and some of them play significant roles during the intestine invasion by this parasite. First, we compared the enzymatic activity of the β-mercaptoethanol-activated recombinant proteases EhCP1, EhCP2, EhCP5, and EhCP112 (rEhCP1, rEhCP2, rEhCP5, and rEhCP112) on Z-Phe-Arg, a specific substrate for L-cathepsin proteases (Ocadiz et al., 2005; Meléndez-López et al., 2007; Quintas-Granados et al., 2009; Hou et al., 2010; Ocadiz-Ruiz et al., 2013; Cornick et al., 2016). All these enzymes degraded the substrate with different efficiency, including papain, the positive control, while BSA, the negative control showed no activity (Figure S1A). Subsequently, we tested the effect of the active enzymes on TEER of confluent Caco-2 cell monolayers. In these experiments, rEhCP112 exhibited the highest effect, dropping TEER by 65% after 90 min interaction with Caco-2 cells (Figure S1B).

**Figure 1:**
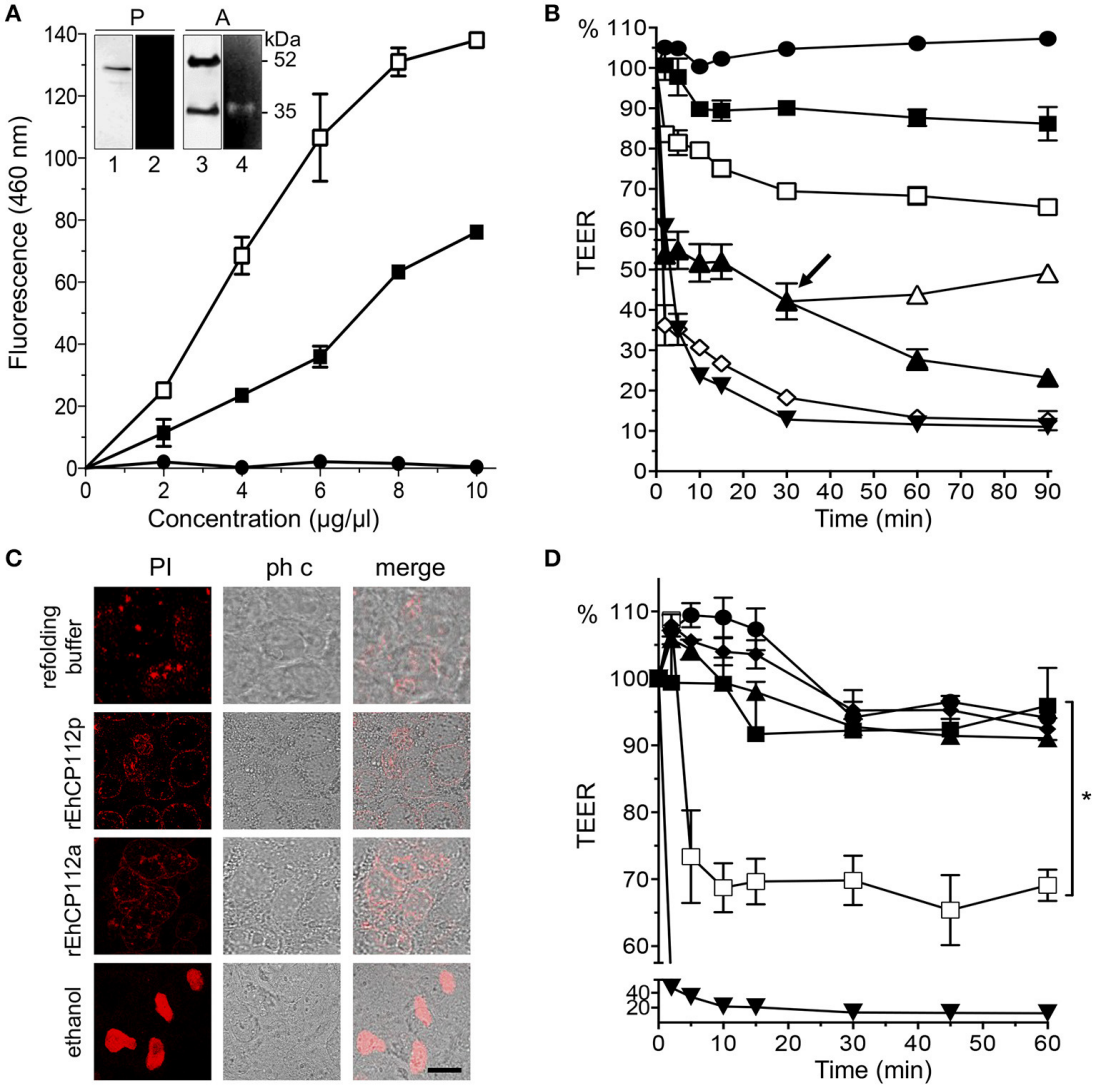
rEhCP112a drops TEER of Caco-2 cell monolayers without membrane disruption. **(A)** (-■-) rEhCP112p or (-□-) rEhCP112a or (-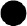-) BSA were incubated with Z-Phe-Arg-AMC and fluorescence was measured at OD_460_ nm. Inset: WB and zymogram assays of (P) rEhCP112p and (A) rEhCP112a, the last one activated by 10 mM β-ME. Lanes 1 and 3: recognition by α-EhCP112 antibody. Lanes 2 and 4: proteolytic activity on gelatin gels. **(B)** Caco-2 cells were incubated with rEhCP112a, (-□-) 10, (-▴-) 20, or (-♢-) 30 μg or with (-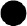-) refolding buffer, or (-■-) rEhCP112p (10 μg) or with (-▾-) trophozoites and then, TEER was measured during 90 min. Arrow signals the time when rEhCP112a was removed (-▴-) and replaced by fresh DMEM and incubated again at 37°C, later, TEER was measured. **(C)** Caco-2 cells incubated for 30 min with rEhCP112a or EhCP112p (20 μg/cm^2^) and then stained with PI and observed by confocal microscopy. Controls included ethanol-fixed Caco-2 cells and cells incubated with the refolding buffer. ph c, phase contrast. Bar = 10 μm. **(D)** rEhCP112a was incubated for 5 min with (-▴-) E-64 or (-■-) α-EhCP112 antibody prior to the incubation with Caco-2 cells or with (-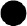-) refolding buffer or with (-□-) rEhCP112a, or with (-■-) rEhCP112p, or with (-▾-) EDTA, then, TEER was measured. TEER values were normalized according to the initial value given by each transwell (1000 Ω/cm^2^). Means and standard errors are represented for each time point of three independent assays performed by triplicate. ^*^p ≤ 0.05.

To explore the molecular mechanism of EhCP112 action without interference by other parasite proteins, we used the purified precursor rEhCP112 (rEhCP112p) and the active protease (rEhCP112a). WB assays revealed the expected 52 and 35 kDa bands corresponding to the precursor and active forms, respectively (Figure 1A). Zymograms evidenced that only the active 35 kDa protein degraded gelatin (Figure 1A, inset). However, both forms degraded the Z-Phe-Arg substrate in a concentration dependent manner (Figure 1A), indicating that rEhCP112p was partially auto-activated after refolding (Ocadiz et al., 2005; Quintas-Granados et al., 2009). rEhCP112a presented higher activity in this assay. Then, we incubated Caco-2 cells with different rEhCP112a concentrations and measured TEER at different times. Results showed that the enzyme caused a drop in TEER in a time and dose-dependent manner (Figure 1B). Live trophozoites (10^5^/cm^2^; 2:1 cells to trophozoites ratio), used as control, decreased the TEER values by 35 and 80% after 2 and 30 min, respectively; similar to the damage produced by 30 μg/cm^2^ of the enzyme (Figure 1B). Next, we explored whether TJs damage compromised the viability of Caco-2 cells and the integrity of the plasma membrane. TEER measurement revealed that cells in permanent contact with the protease presented a continuous drop in TEER, whereas cells in which rEhCP112a was removed, gradually recovered TEER (Figure 1B, arrow). Of note, a 30 min incubation with the active protease did not lead to cell death in Caco-2 cells (Figure 1C). These findings highlight that at this time and dose, the drop in TEER produced by the enzyme was reversible and did neither compromise membrane integrity nor cell viability. Incubation of rEhCP112a (10 μg/cm^2^) with the α-EhCP112 antibody or E-64 protease inhibitor, before being added to the Caco-2 cells, prevented the drop in TEER (Figure 1D), evidencing the specific effect of EhCP112 on TJs.

### Overexpression of EhCP112 in trophozoites increases TEER dropping

**Figure 2:**
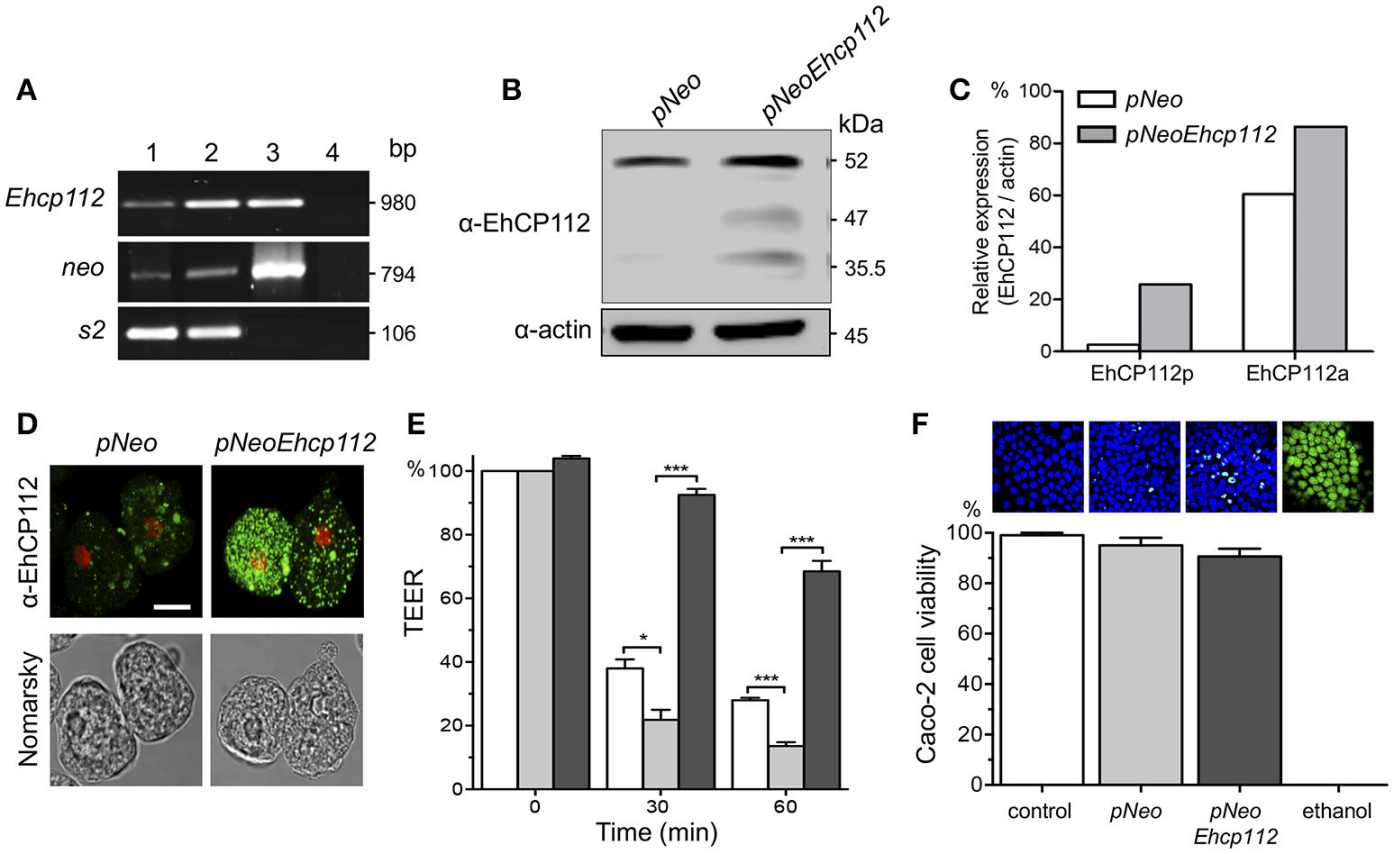
EhCP112 overexpression increases TEER dropping without affecting cell viability. **(A)** RT-PCR analysis of stably-transfected trophozoites with (lane 1) *pNeo* or (lane 2) *pNeoEhcp112* construction; (lane 3) *pNeoEhcp112* plasmid; and (lane 4) PCR mixture without DNA, showing the *neo* and s2 control transcripts. **(B)** *pNeoEhcp112* and *pNeo* transfected trophozoites were lysed and submitted to WB and revealed using the antibodies indicated at left. **(C)** Densitometry analysis of the data shown in **(B)**, using actin as control. **(D)** Immunofluorescence assays of *pNeoEhcp112* or *pNeo* transfected trophozoites incubated with α-EhCP112 and secondary-FITC antibodies. Nuclei were stained with PI. Bar = 10 μm. **(E)** Caco-2 cells were incubated with (□) *pNeo* or (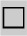) *pNeoEhcp112* or (■) *pNeoEhcp112* transfected trophozoites pre-incubated with α-EhCP112 antibody and TEER was measured at 30 and 60 min. TEER values were normalized according to the initial value given by each transwell (1,000 Ω/cm^2^). Means and standard errors are represented for each time point of an assay performed by triplicate. ^*^p ≤ 0.01; ^***^p ≤ 0.001. **(F)** Caco-2 cells viability was measured by Sytox reagent. Upper panels show images of the Sytox treated cell monolayers.

To further corroborate the EhCP112 effect on gate functions, we generated trophozoites stably overexpressing *pNeoEhcp112*. RT-PCR confirmed an increase of the *ehcp112* transcript in *pNeoEhcp112*-transfected trophozoites in comparison with the control *pNeo* (Figure 2A). WB assays showed higher amounts of the different protease protein forms, including the precursor and active enzyme, in comparison to the control (Figures 2B,C). Confocal microscopy images also showed more EhCP112 in the cytoplasm (Figure 2D). EhCP112-overexpressing trophozoites caused a significantly higher drop in TEER in Caco-2 cells than trophozoites transfected with *pNeo* only. Antibodies against EhCP112 significantly prevented the TEER decrease (Figure 2E). The effect on TEER correlated with the increase of the enzyme in transfected trophozoites. However, to corroborate that the drop in TEER was not directly related to cellular damage at the time tested, we quantified cell viability and confirmed that 98% of Caco-2 cells were still viable after treatment with trophozoites (Figure 2F).

### rEhCP112a does not affect paracellular permeability of macromolecules

**Figure 3:**
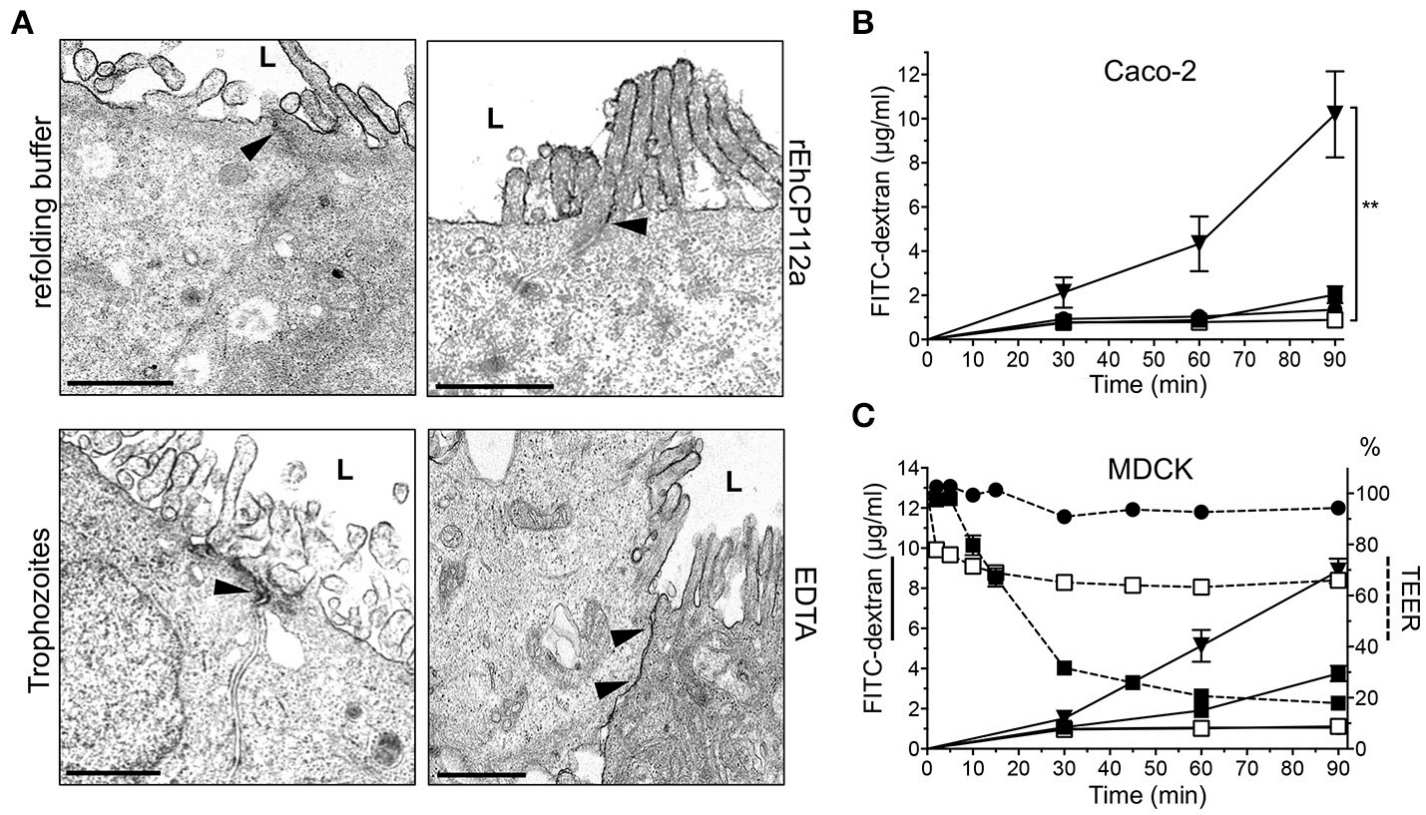
rEhCP112a does not affect the macromolecule flux in Caco-2 and MDCK cells. **(A)** TEM of Caco-2 cells incubated for 30 min with 10 μg/cm^2^ of rEhCP112a, refolding buffer, EDTA or live trophozoites (10^5^, 10 min), and then, glutaraldehyde fixed and stained with RR marker. L: lumen or apical side. Arrowheads: RR label at intercellular space. Bar: 1μm. **(B)** FITC-dextran (2–4 kDa, 5 μg/ml) was added to the apical chamber of Caco-2 cells grown in transwells and incubated with (-□-) rEhCP112a, or (-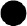-) refolding buffer, or (-■-) live trophozoites, or (-▾-) EDTA. FITC-dextran was obtained from the basal chamber and measured by fluorescence spectroscopy at OD_520_ nm. **(C)** MDCK cells were grown in transwells and incubated with (-□-) rEhCP112a, or (-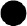-) refolding buffer, or (-■-) live trophozoites, or (-▾-) EDTA, then, FITC dextran and TEER values were measured. Data represent means and standard errors of three independent assays. ^**^p ≤ 0.01.

Gate function comprises control of both ion and macromolecular flux (Liang and Weber, 2014). To investigate whether rEhCP112a enzyme also disturbs the paracellular permeability for macromolecules, we performed TEM assays using RR dye, an electrodense marker. TEM images revealed that RR did not enter into the intercellular space in Caco-2 monolayers incubated with the active enzyme (Figure 3A). In addition, TEM images of cell monolayers in contact with trophozoites evidenced that RR was also restricted to the apical side of Caco-2 cells (Figure 3A). As expected, EDTA, used as positive control, allowed RR diffusion indicating opening of junctions (Gonzalez-Mariscal et al., 1985; Figure 3A). FITC-dextran (2–4 kDa) did not diffuse either through the intercellular space in Caco-2 cells treated with rEhCP112 or trophozoites (Figure 3B); further indicating that the macromolecular flux was not altered by the enzyme.

In contrast to our findings with Caco-2 cells, earlier studies demonstrated that RR penetrates through the paracellular space in MDCK cell monolayers incubated with live trophozoites (Martinez-Palomo et al., 1985). Thus, we evaluated the effect of rEhCP112 on MDCK cell monolayers. Our results showed that, in agreement to the results reported by Martinez-Palomo et al. (1985), but unlike our data in Caco-2 cells, macromolecular flux in MDCK cells increased after incubation with trophozoites (Figure 3C). By contrast, rEhCP112a had no effect on MDCK paracellular flux, but also increased ion flux as in Caco-2 cells (Figures 1B, 3C). These results show that *E. histolytica* trophozoites have differential effects on different types of epithelia. It is currently unknown what causes these discrepancies, but it is likely that differential expression of TJ proteins plays a role (Elkouby-Naor and Ben-Yosef, 2010; Lu et al., 2013).

### rEhCP112a disrupts fence function

**Figure 4:**
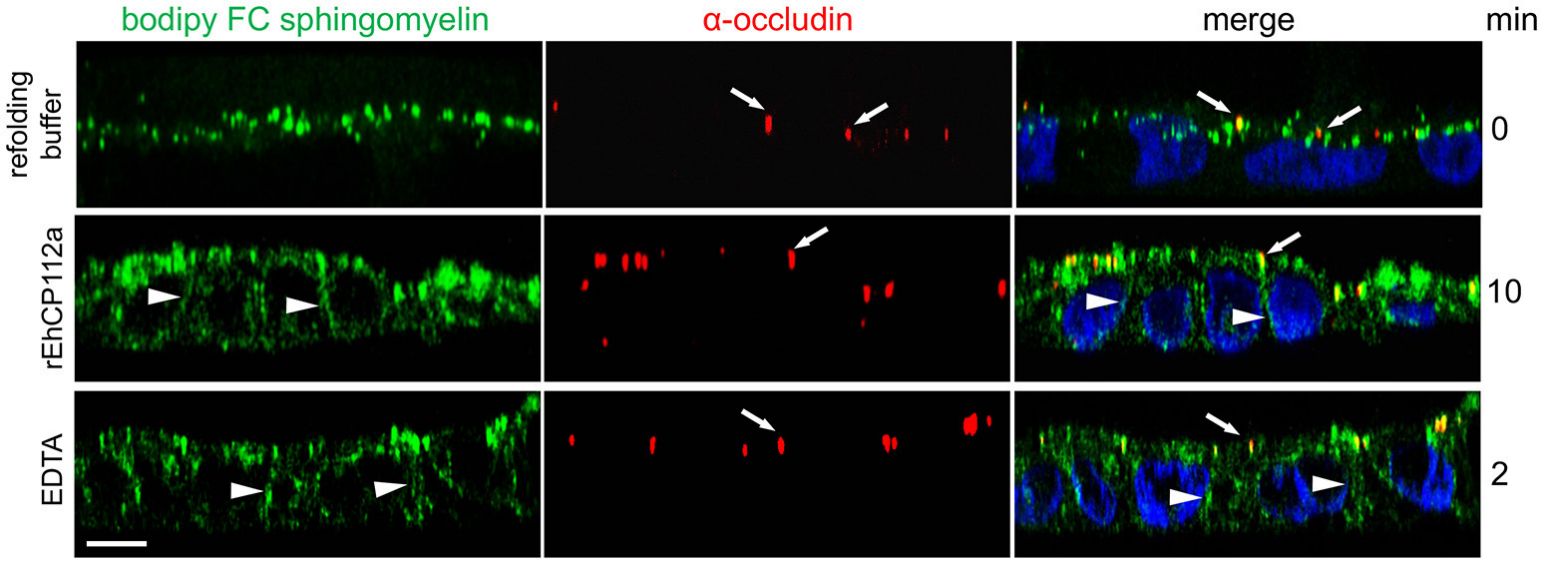
rEhCP112a provokes bodipy diffusion in Caco-2 cells. Caco-2 cells were apically labeled by bodipy FC sphingomyelin (green) and incubated with rEhCP112a or EDTA or refolding buffer. Cells were fixed and incubated with α-occludin antibody (red) as a TJs marker, then, cells were observed through confocal microscope in the *zy*-plane. Nuclei (blue) were counterstained with DAPI. Arrowheads: lipid diffused to the basolateral membranes. Arrows: occludin at TJs. Bar: 10 μm.

In addition to the gate function, TJs retain plasma membrane molecules in either the apical or basolateral compartment, thus maintaining cell polarization (fence function; Zihni et al., 2016). When the fence function is disrupted, proteins and lipids that constitute the apical and basolateral membranes diffuse freely. To explore the effect of EhCP112 on fence function in Caco-2 cells, apical lipids were stained using fluorescently-labeled Bodipy FC-Sphingomyelin, incubated with rEhCP112a, and examined by confocal microscopy. We found that after 10 min, rEhCP112a caused diffusion of sphingomyelin to the basolateral membrane implying disruption of the fence function (Figure 4). Cells treated with the refolding buffer as negative control, indeed maintained the lipid at the apical membrane. Occludin did not shift significantly to the basolateral region after rEhCP112a treatment (Figure 4), strengthening the hypothesis that this TJ protein is not affected by EhCP112. These results indicate that rEhCP112 affects ion flux and fence functions of TJs.

### rEhCP112a enters via the paracellular route

**Figure 5:**
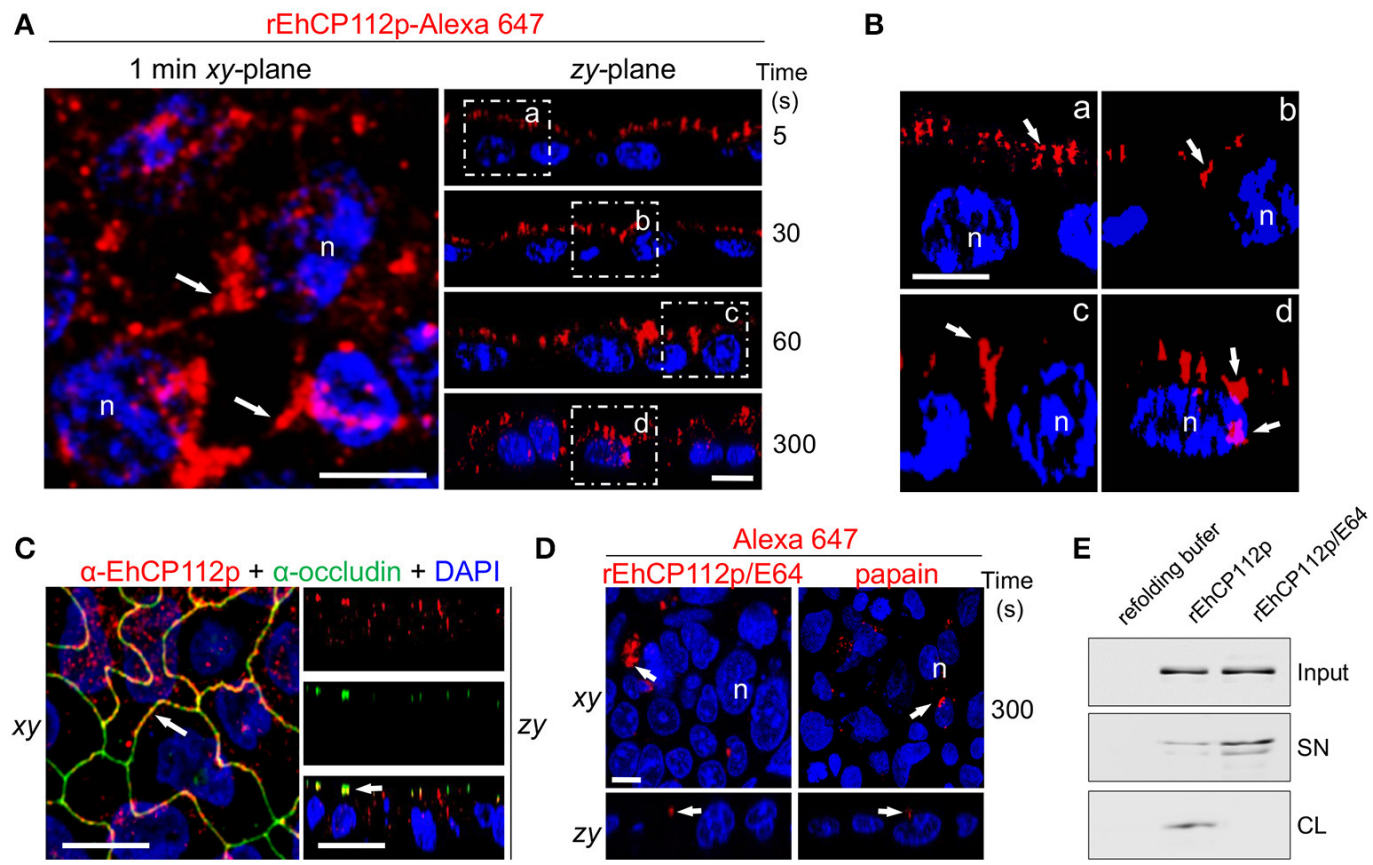
rEhCP112a enters to Caco-2 cell monolayers through the intercellular space. **(A)** rEhCP112p coupled to Alexa 647 (red) was apically added to Caco-2 cell monolayers for different times, then, cells were fixed and analyzed through confocal microscope in the *xy*- and *zy*-planes. Square areas are magnified in **(B). (C)** Caco-2 cells treated as in **(A)** were incubated with α-occludin antibody. **(D)** As in **(A)**, but rEhCP112p was pre-incubated with E-64. Labeled papain was used as control. Nuclei (n) were counterstained with DAPI (blue). Arrows: labeled enzymes. Bar: 10 μm. **(E)** WB assays of recovered supernatants (SN) from Caco-2 cells treated with refolding buffer or rEhCP112p or rEhCP112p-E-64. Input: enzymes added to epithelial cells. CL, cell lysates.

Some pathogens provoke delocalization or degradation of TJ proteins, affecting gate and fence functions (Sousa et al., 2005; Hodges and Gill, 2010; Maia-Brigagao et al., 2012). Based on these findings, we analyzed the route that Alexa 647-labeled rEhCP112 follows after its contact with the apical side of Caco-2 cells. In these experiments we employed the precursor form of the enzyme, to slower the degradation process. Confocal microscopy revealed that after 5 s of interaction, rEhCP112p was already distributed along the apical cell surface (Figure 3A). This was more evident in zy-plane images (Figures 5A,B). At 30 s, rEhCP112p appeared concentrated in the apical region between two cells, but remained outside of the cells. After 1 min of incubation, fluorescent rEhCP112p was present within the intercellular space, indicating its affinity for the paracellular route. After 5 min, rEhCP112p still appeared along the lateral membrane and now some of the enzyme already entered the cells (Figures 5A,B). Moreover, we detected co-localization of the protease with occludin at TJ corroborating that EhCP112 indeed interacts with TJs (Figure 5C). We explored whether the inactivated enzyme was also able to bind to and penetrate into the cells, but only detected a very poor signal along the apical surface of Caco-2 cell monolayers suggesting that enzyme activity could be important for both binding to the apical surface and subsequent paracellular entry (Figure 5D). To confirm this, we recovered the supernatants of Caco-2 cells incubated with rEhCP112p and rEhCP112p pre-treated with E-64. WB assays showed that EhCP112p-E-64 remained in the supernatant, whereas EhCP112p amount was lower because the enzyme bound to Caco-2 cells (Figure 5E). These Results don't discard that the enzyme could also follow the transcellular route to penetrate the Caco-2 cell monolayers. Intriguingly, images showed the rEhCP112 entrance into the cell after 5 min of interaction (Figure 5A); however, IP staining (Figure 1C) revealed no membrane damage. This could be explained by the fact that EhCP112 is also internalized by caveolae and clathrin coated vesicles as shown recently by Hernández-Nava et al. (2017) in MDCK cell monolayers.

### rEhCP112a delocalizes claudin-1 and claudin-2 but does not affect occludin or ZO-1

**Figure 6:**
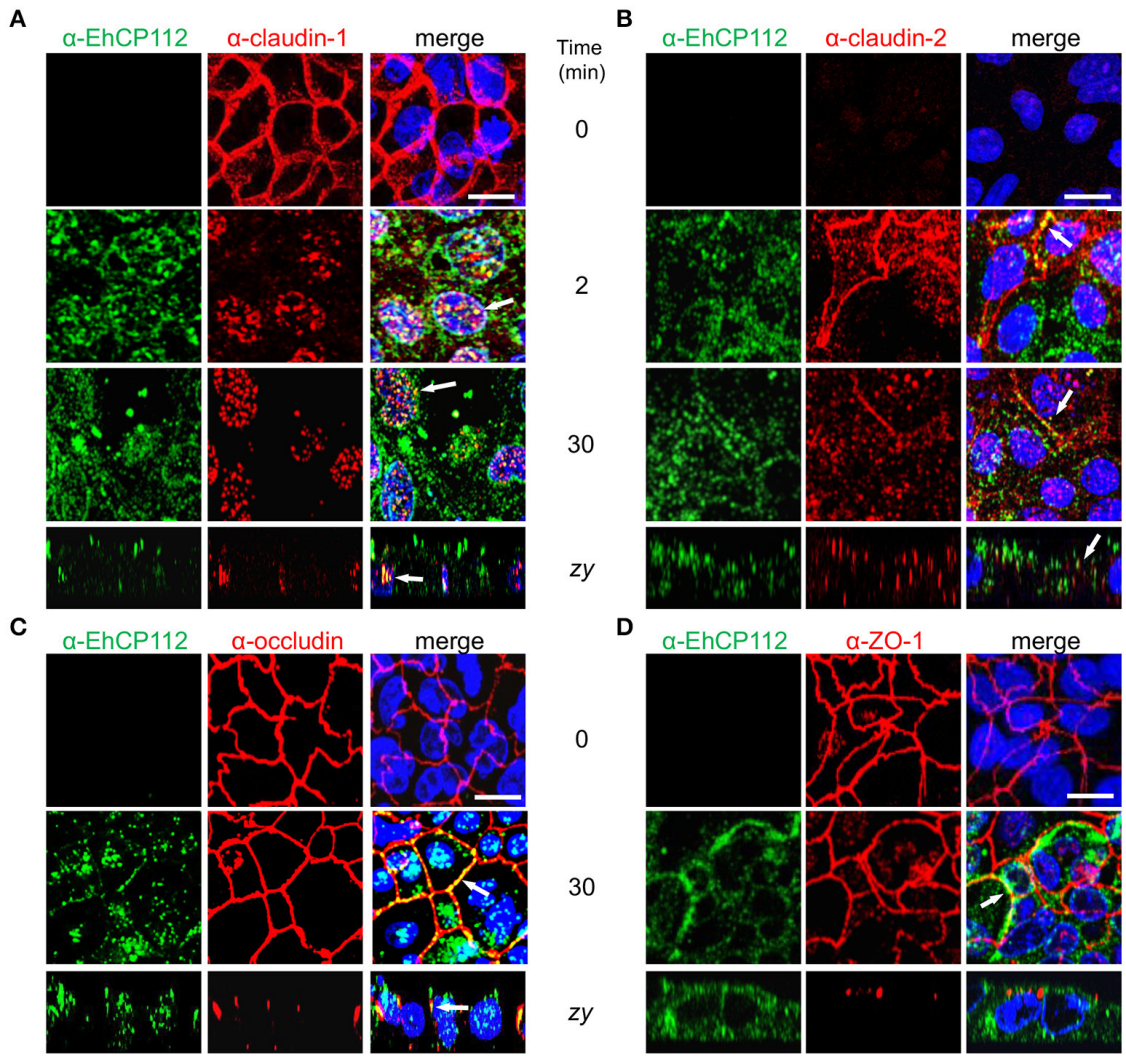
rEhCP112a alters claudin-1 and claudin-2 localization. **(A–D)** Confocal microscopy of Caco-2 cell monolayers incubated with rEhCP112a for 0, 2, or 30 min and then, incubated with α-EhCP112 (green) and **(A)** α-claudin-1 (red) or **(B)** α-claudin-2 (red), or **(C)** α-occludin (red) or **(D)** α-ZO-1 (red) antibodies, followed by the corresponding secondary antibodies. Nuclei were counterstained with DAPI. Arrows: co-localization. Bar: 10 μm.

After knowing that the intercellular space is a pathway followed by EhCP112 to penetrate the epithelium, we studied the effect of the enzyme on the cellular location of TJ proteins until 30 min of incubation with rEhCP112a. Confluent Caco-2 cells were incubated with rEhCP112a for different times and then, by specific antibodies, the cellular location of claudin-1, claudin-2, occludin, and ZO-1 were analyzed through confocal microscopy. In the absence of EhCP112, the cellular proteins analyzed, except claudin-2, presented the typical honeycomb pattern already described for TJ proteins. However, after 2 min of incubation with the enzyme, claudin-1 moved to the cytoplasm and nucleus, co-localizing in some points with rEhCP112a (Figure 6A). The translocation of claudin-1 to the nucleus is documented in colon cells, after certain stimuli and metastatic transformation (Dhawan et al., 2005). In agreement with many reports on the scarce presence of claudin-2 in tight epithelia (Escaffit et al., 2005), it gave very faint signal in control confluent Caco-2 cells. Interestingly, at 2 min of incubation with EhCP112, as it has been well documented for sparse cell cultures (Escaffit et al., 2005), when the cells forming the monolayer began to loosen due to the effect of the rEhCP112a protease, fluorescence of claudin-2 increased at the cellular borders and in the cytoplasm, co-localizing in some points with the enzyme. Later, at 30 min, claudin-2 decreased again, probably due to the degradative effect of the enzyme (Figure 6B). In contrast, occludin and ZO-1 patterns appeared without changes after 30 min of interaction (Figures 6C,D), suggesting that EhCP112 has effect only on claudins. The unaltered occludin pattern is in concordance with the unaffected macromolecules flux after the enzyme treatment showed in Figure 5.

### rEhCP112a affects claudin-1 and claudin-2 integrity, but not claudin-4, occludin, ZO-1, and ZO-2

**Figure 7:**
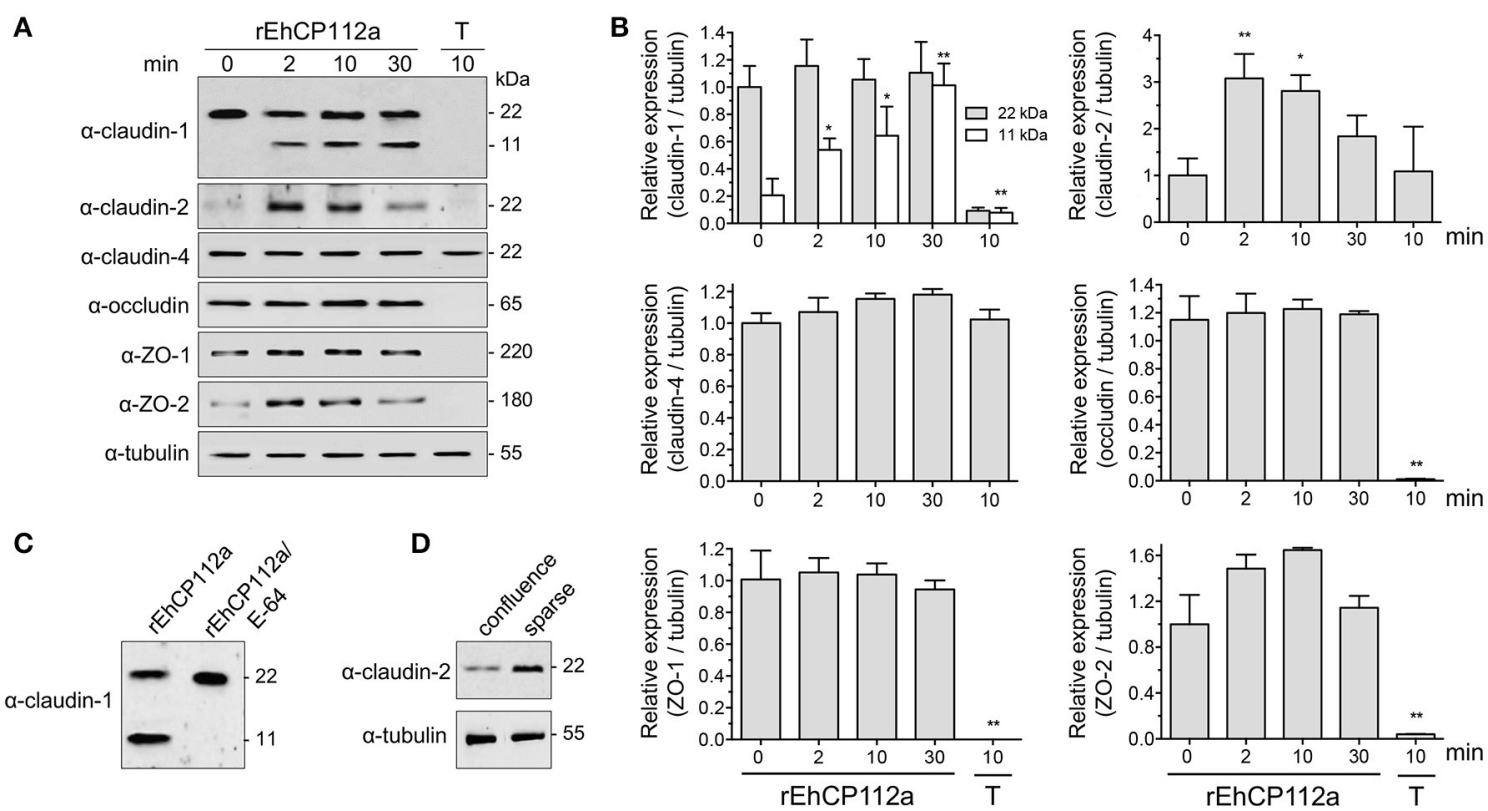
rEhCP112a degrades claudin-1 and increases claudin-2 expression in Caco-2 cells. **(A)** Caco-2 cells incubated with rEhCP112a for different times or with live trophozoites (T) for 10 min, submitted to WB using antibodies against TJ proteins indicated at left and α-tubulin as a loading control. **(B)** Densitometry analysis of bands obtained in **(A)** for each TJs protein with regards to tubulin. ^*^p ≤ 0.05, ^**^p ≤ 0.001 compared to the basal condition (0 time). **(C)** Caco-2 cells incubated with rEhCP112a or E-64-pre-treated rEhCP112a, submitted to WB using α-claudin-1 antibody. **(D)** Caco-2 cells lysates from confluent and sparse cultures and submitted to WB using antibodies indicated at left. Blots are representative of at least three independent experiments.

To explore the effect of rEhCP112a on TJ proteins integrity, confluent Caco-2 cells incubated with rEhCP112a were lysed and submitted to WB assays. The α-claudin-1 antibody recognized a 22 kDa band in control cells. After incubation with rEhCP112a, an 11 kDa band appeared and gradually increased upon time, suggesting degradation of claudin-1 (Figures 7A,B). To obtain more evidence on the claudin-1 degradation after contact with the protease, we treated rEhCP112a with E-64 inhibitor, before the enzyme addition to Caco-2-cells. In these experiments, the 11 kDa band did not show up, substantiating that it could be a degradation product due to the enzyme action (Figure 7C). However, the 22 kDa band maintained similar amount through the experiments, possibly due to a compensatory *de novo* synthesis of the protein, but this assumption needs to be experimentally proved. Similarly to confocal immunolocalization assays (Figure 6B), claudin-2 in confluent Caco-2 cell monolayers was scarce, but 2 min after incubation with rEhCP112a, when the protease has started to act on TJs, fluorescence corresponding to claudin-2 increased. At 30 min incubation the recognition of claudin-2 by the antibody, decreased (Figures 7A,B), probably due to the degradative effect of the continuous enzyme exposure. We confirmed by WB assays that claudin-2 is in higher amount in confluent Caco-2 cell cultures than in sparse cells that were not in contact with the enzyme (Figure 7D). rEhCP112a did not affect claudin-4, occludin, ZO-1, and ZO-2 (Figures 7A,B). Trophozoites degraded all proteins tested except claudin-4 and tubulin (Figures 7A,B), confirming that distinct trophozoites molecules produce dissimilar effect on host cell proteins. Intriguingly, trophozoites degraded occludin; although they did not affect paracellular flux in Caco-2 cells (Figure 3), possibly, claudin-4, a molecule that also regulates paracellular flux (Takizawa et al., 2014; Khan and Asif, 2015) performed a compensatory effect in this function, given that it was not affected by trophozoites.

### rEhCP112a associates to TJ proteins

**Figure 8:**
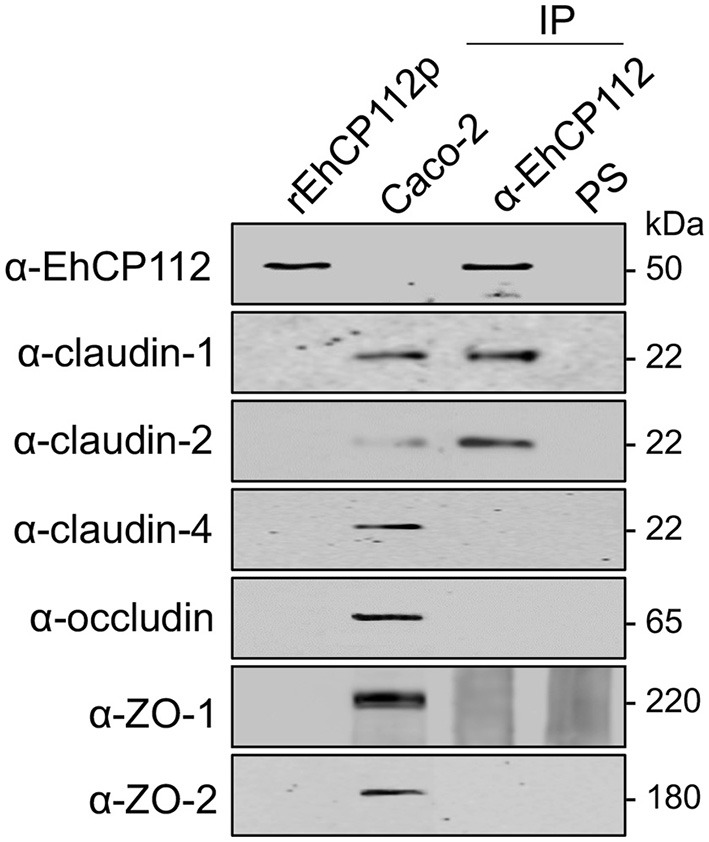
rEhCP112p binds to claudin-1 and claudin-2. Confluent Caco-2 cell monolayers were incubated for 10 min with rEhCP112p, then, cell lysates were immunoprecipitated (IP) in the presence of protease inhibitors, using α-EhCP112 antibody or pre-immune serum (PS). Immunoprecipitates, rEhCP112p and Caco-2 cell lysates (Caco-2) were submitted to WB assays and revealed using the antibodies indicated at left.

The immunofluorescence experiments using Caco-2 cell monolayers and rEhCP112a showed that the enzyme affects claudin-1 and claudin-2, co-localizing with rEhCP112 and delocalizing them from their initial site in the cell. Therefore, to investigate the association of TJ proteins with the enzyme, we assessed immunoprecipitation assays employing the α-EhCP112 antibody. In these experiments, we used rEhCP112p to slow the proteolytic process; and, immediately after the immunoprecipitation with the antibody, we added protease inhibitors, to avoid proteolytic activity. In WB assays of the immunoprecipitates, the specific antibodies detected claudin-1 and claudin-2, but not claudin-4, occludin, ZO-1, and ZO-2 (Figure 8). Similarly to the immunofluorescence and protein degradation assays (Figures 6, 7), these data reinforce the assumption that claudin-1 and claudin-2 are binding targets for EhCP112 during the trophozoite attack. Again, recognition of claudin-2 in the immunoprecipitates was higher than in cells that were not in contact with the protease (Figure 8), suggesting that the interaction of the cells with rEhCP112p provoked an enrichment of this protein, in agreement with results obtained in Figures 6, 7.

### rEhCP112a impairs colonic epithelium in C57/BL6 mice

**Figure 9:**
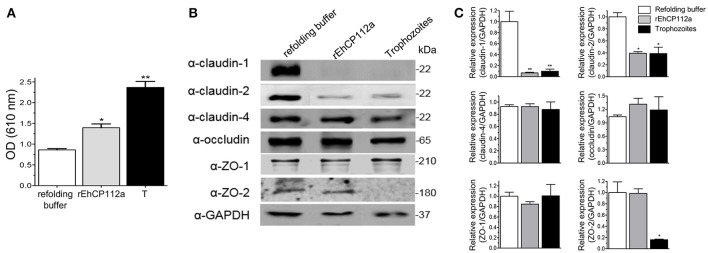
rEhCP112a impairs epithelial barrier and degrades claudin-1 and claudin-2 in colonic epithelium of mice. C57/BL6 mice were rectally inoculated with 50 μg of rEhCP112a or with live trophozoites (10^6^) or with refolding buffer and incubated for 30 min (*n* = 6). **(A)** Evan's blue permeability was spectrophotometrically measured at OD_610nm_. **(B)** WB assays of epithelial colon of mice treated with rEhCP112a (50 μg in 300 μl of refolding buffer), or live trophozoites (10^6^ in 300 μl of refolding buffer), or refolding buffer (300 μl). Proteins were revealed by antibodies indicated at left. α-GAPDH antibody was used as loading control. **(C)** Densitometry analysis of bands in **(B)**. ^*^p ≤ 0.05, ^**^p ≤ 0.001. Data were compared to the control.

The TJ gate function in MDCK and Caco-2 cells suffered differential impact after the trophozoites and EhCP112 attack (Figure 3), supporting the distinct susceptibility of epithelial cell lines to trophozoites molecules. In *in vivo* studies, we need to have in mind that the host immune response plays a double role, modulating the damage that the parasite and its molecules produce (Petri, 2008; Mortimer and Chadee, 2010) and generating defense cells and molecules that could contribute to the tissue injure. To gain further insight on the *E. histolytica* damage produced on animal models, we investigated the effect of rEhCP112a on C57/BL6 mice, a strain susceptible to the trophozoites colonization (Kissoon-Singh et al., 2013). In this model, we used Evan's blue tracer to determine whether rEhCP112a alters the intestinal permeability (Lange et al., 1994). Results showed that, while in control mice, treated only with the re-folding buffer no evident damage was observed in the tissues, but 30 min after inoculation of 50 μg of the enzyme resuspended in the refolding buffer, the Evan's blue tracer penetrated the mouse intestinal epithelium. Permeability of rEhCP112a-treated mice, increased twice, in comparison with refolding buffer-treated animals (Figure 9A). On the other hand, the epithelium of mice inoculated with 10^6^ trophozoites, absorbed twice Evan's blue than the rEhCP112a-treated animals, indicating that trophozoites had a higher effect on epithelial permeability.

### rEhCP112a alters claudin-1 and claudin-2 in colonic epithelium of C57/BL6 mice

Our results using Caco-2 cells evidenced important alterations of claudin-1 and claudin-2 due to the effect of rEhCP112a, thus, we analyzed the integrity of TJ proteins in the mouse colonic epithelium after inoculation with the protease. In agreement with results found in experiments with Caco-2 cells monolayers (Figure 7), WB assays using epithelia obtained from rEhCP112a-inoculated mice revealed that claudin-1 and claudin-2 were degraded, whereas, claudin-4, occludin, ZO-1, and ZO-2, as well as the control GAPDH, appeared without modification (Figures 9B,C). Trophozoites, had the same effect than rEhCP112a, but they also degraded ZO-2 and, in contrast with the Caco-2 cells, we did not observe degradation of occludin and ZO-1. In colonic epithelium, claudin-2 is restricted to the proliferative regions of the intestine, but is poorly expressed in differentiated cells (Escaffit et al., 2005). Here, we used colonic tissue samples containing both regions of the whole epithelium (Figures 9B,C).

**Figure 10:**
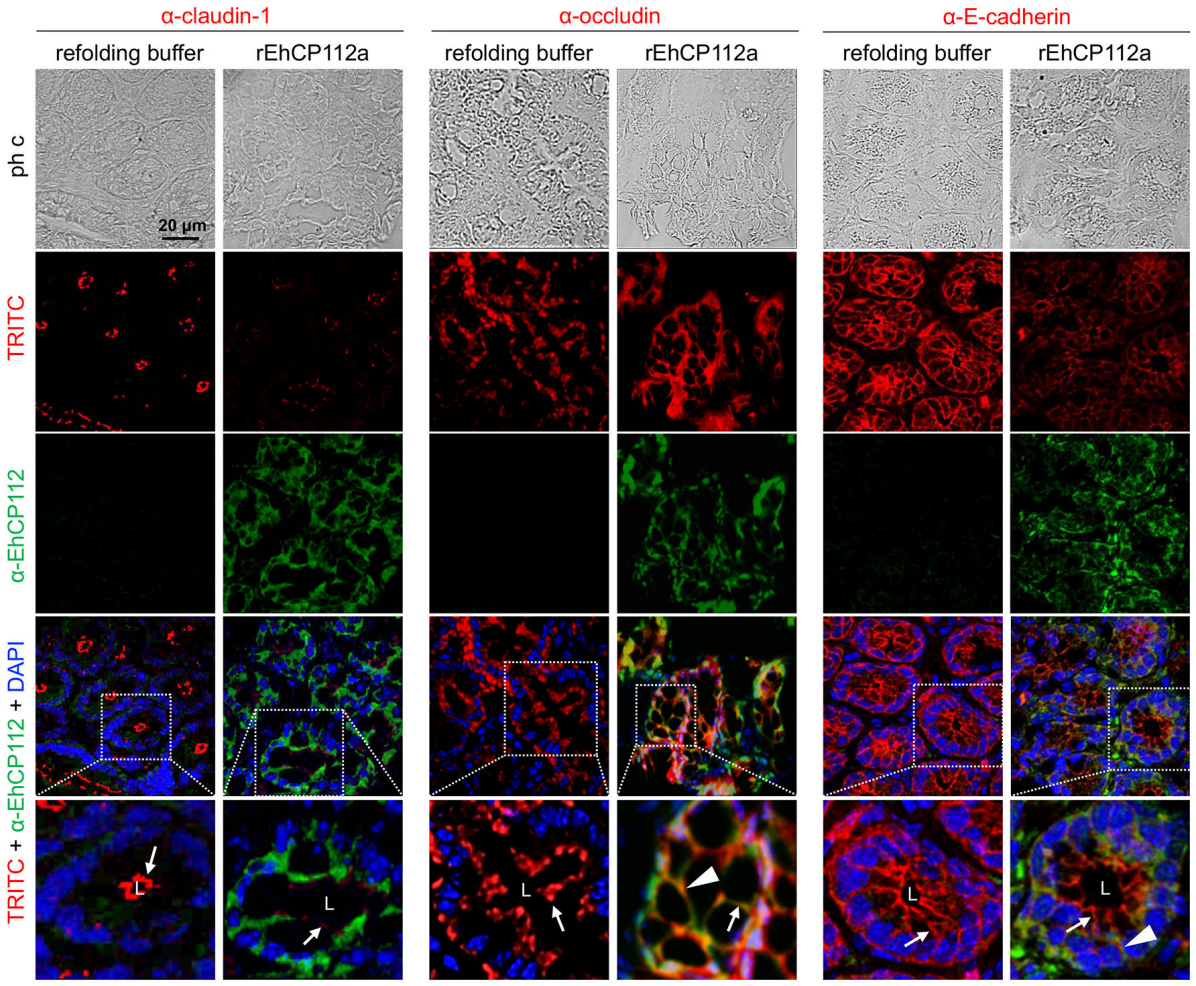
rEhCP112a alters claudin-1 in colonic epithelium of mice. C57/BL6 mice were treated as in Figure 9. Frozen tissue sections of were incubated with α-EhCP112 (green) and α-claudin-1 (red), or α-occludin (red), or α-E-cadherin (red) antibodies and analyzed by confocal microscopy in the *xy*-plane. Nuclei were counterstained with DAPI. Arrowheads: claudin-1 localization in TJs. White squares areas were magnified in the bottom row. Arrows: localization of the proteins at cellular borders. Arrowheads: co-localization of EhCP112 with occludin or E-cadherin. L, lumen.

To better examine the damage produced by the enzyme on the TJ proteins of intestinal epithelium, we performed confocal microscopy assays to localize claudin-1 and occludin in treated and untreated mice. Images of colon sections using α-EhCP112 and α-claudin-1 antibodies showed claudin-1 at the apical side, along the colonic epithelium of control mice. In concordance with the WB results, claudin-1 significantly decreased in rEhCP112a-treated mice and the enzyme appeared at the cellular borders and in the cytoplasm of epithelial cells (Figure 10). Occludin, localized also at cellular borders, was not altered after rEhCP112 incubation, accordingly to WB results (Figure 9B). E-cadherin, located along cellular borders of epithelial cells, below TJs, was used as a protein control damaged by rEhCP112 in intestinal epithelial cells (Hernández-Nava et al., 2017). In these experiments E-cadherin also diminished after incubation of intestinal epithelium with the protease (Figure 10).

Finally, after knowing that rEhCP112 was able to damage the intestinal epithelium, we wondered whether the enzyme was able to cross the mucosa layer that protects the intestinal epithelium. We performed confocal microscopy assays of the intestinal epithelium from rEhCP112a-treated mice, using α-mucin-2 and α-EhCP112 antibodies. Images showed that EhCP112 appeared alone in the luminal side of the intestine. Besides, α-EhCP112 antibodies also illuminated areas where mucin was abundant and decorated the epithelium, where goblet cells were present (Figure S2). Both proteins appeared close each other in the epithelium (Figure S2).

In conclusion, our results demonstrate that EhCP112 damages cultured epithelial cells and the mouse intestinal epithelium, disturbing TJs through a direct effect on claudin-1 and claudin-2. Our findings reveal a crucial role of EhCP112 enzyme during invasion of *E. histolytica* to the host cells and point out the potential of this molecule as a therapeutic target.

## Discussion

**Figure 11:**
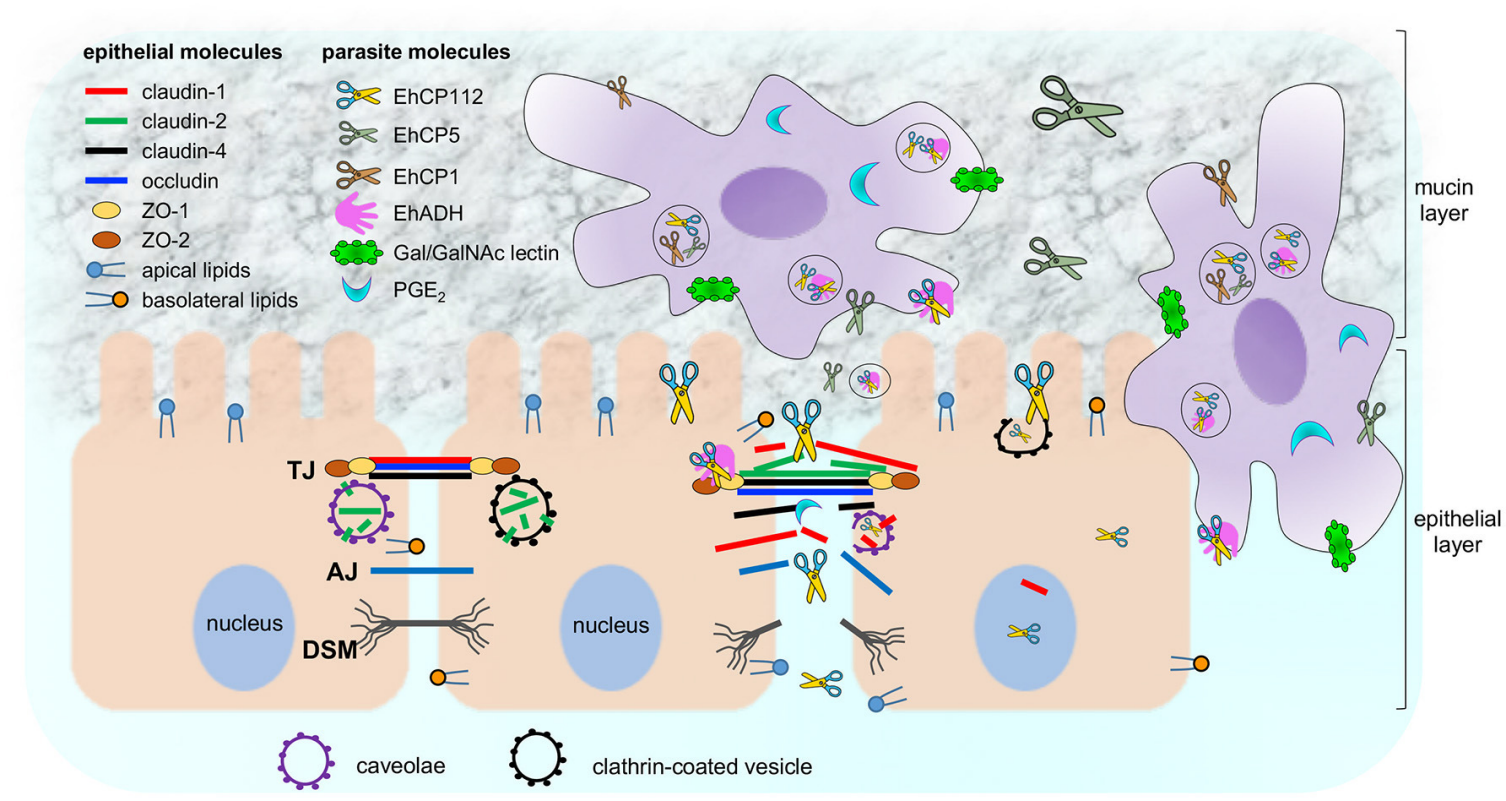
Working model of *E. histolytica* damage on epithelium focused on EhCP112 action. Trophozoites secrete different molecules (PGE_2_ and cystein proteases) to cross the mucin layer and reach the epithelium. Then, trophozoites adhere to epithelial cells by specific molecules (Gal/GalNAc, EhADH, and others). EhCP112 reaches the intercellular space, selectively affecting claudin-1 and claudin-2, but not claudin-4, occludin, ZO-1 and ZO-2 that are damaged by other trophozoites molecules. TJs are disrupted, affecting gate and fence functions. Then, EhCP112 moves to AJs and DSMs. The cell-cell contact is lost and epithelial cells detach, facilitating the invasion by trophozoites. Alternatively, EhCP112 is internalized to the cell by caveolae and clathrin coated pits, contributing to epithelial damage through protein degradation.

*E. histolytica* is a protozoan with a large arsenal of virulence molecules; among them, the CPs (Figure 11). The majority of the studied CPs is localized in cytoplasm and membranes, and, some are differentially expressed after contact of trophozoites with target cells (Thibeaux et al., 2013). In this work we analyzed the molecular events that EhCP112 follows to damage epithelial cells, impairing TJ functions, degrading and delocalizing claudin-1 and claudin-2, and then, at longer times, probably, disrupting the epithelium. The study of the mechanisms and pathways that virulence molecules follow to damage host tissues is essential to understand the parasite pathogenicity and to defeat amoebiasis, by designing better diagnosis methods and finding vaccine candidates.

Before starting our experiments with the purified rEhCP12, we analyzed the comparative activity of the four main CPs studied in *E. histolytica*, producing the recombinant enzymes to separately scrutinize their effect on TJs (Figure S1). However, we are aware that in addition to the damage that they cause to TJs, these EhCPs have other cellular functions related to the host harm production, such as digestion of mucin, collagen, fibronectin, activation of the inflammasome, and others. EhCP112 yielded the major TEER dropping on Caco-2 cells, but EhCP1, EhCP2, and EhCP5 also affected TEER in different degree. This substantiated that these enzymes and other virulence molecules sum up their action during epithelial invasion and destruction. For example, pre-incubation of trophozoites with serine protease inhibitors also prevent TEER dropping of epithelial cells (Lauwaet et al., 2004), evidencing the participation of *E. histolytica* serine proteases in TJs disruption. Additionally, PGE_2_, a molecule secreted by *E. histolytica*, also drops TEER (Lejeune et al., 2011).

Two facts showed here, confirmed the specific action of EhCP112 on TEER: (i) the higher effect on Caco-2 confluent cells produced by trophozoites overexpressing the enzyme; and (ii) the prevention of TEER dropping by protease inhibitors and the α-EhCP112 antibody. Interestingly, damage produced by EhCP112 on TJs was not reflected as irreversible cell injure after 30 min, even when TEER was dropped in more than 50%. The recovering of the initial TEER values in Caco-2 cultures after removing the enzyme, indicated that cells were not irreversible damaged by rEhCP112; and impermeability to PI, a vital dye, denoted membrane integrity. *Toxoplama gondii* and *G. duodenalis* also provoke TEER dropping, (Maia-Brigagao et al., 2012; Briceno et al., 2016) but it is still unknown which parasite molecules are responsible for this event, and damage reversibility has not been studied. rEhCP112p is able to auto-activate (Figure 1A; Ocadiz et al., 2005; Quintas-Granados et al., 2009). Thus, we wonder how the enzyme can be in the cytoplasm without devastating the trophozoites. We speculate that trophozoites possess a mechanism to regulate activation of EhCP112, avoiding potential self-damage by the active protease. Other possibility is that the enzymes could be in small vesicles or vacuoles that we did not detect here. Further experiments are needed to elucidate this.

TJs have several functions, among them the ion and macromolecules flux control and the fence function. TEER is directly related to the ion flux controlled by claudin-1 and claudin-2, whereas, occludin is mainly related to the macromolecules flux regulation. Our results proved that rEhCP112 has no effect in macromolecules flux of either Caco-2 cells or MDCK cell monolayers. Interestingly, trophozoites affected it on MDCK but not in Caco-2 cells. This is other evidence on the participation of several molecules in damage production by trophozoites and the diverse susceptibility of distinct epithelia to their action, which might be related to the dissimilar TJs composition in Caco-2 and MDCK cell monolayers (Elkouby-Naor and Ben-Yosef, 2010; Lu et al., 2013).

Bodipy FC sphingomyelin labeling assays allowed us to evaluate TJs fence function (Figure 4). Fence function maintains cell polarity and is controlled also by claudin-1. The fence dysfunction provoked by rEhCP112 caused a lipid shift from the apical to the basolateral membrane of Caco-2 cells. The enzyme migration is accompanied by a host cell protein and lipids delocalization, which is better observed in the co-localization assays using α-TJs together with α-rEhCP112 antibodies (Figure 6). Lipid and protein delocalization might change the position of certain cell receptors that the parasite could use during invasion, exposing them for protease action. The very short time needed by rEhCP112p to bind to and penetrate the cell suggests the affinity of the protease for membrane molecules. In fact, in trophozoites, the EhCPADH serves as a receptor for erythrocytes and epithelial cells (Rodriguez et al., 1989). After contact, activation could be self-provoked or triggered by unknown host cell molecules. Recently, we reported that the enzyme is also internalized by clathrin and caveolin covered pits (Hernández-Nava et al., 2017). In concordance, it is clear that the cytoplasmic access of EhCP112 occurred without plasma membrane disruption (Figure 1). It was also interesting to reveal that if we blocked the protease active site by E-64, the enzyme did not adhere to the cells (Figure 5), pointing out the importance of the active site, not only in the protease action, but also in its interaction with host cell proteins. It is possible that this could be related to the protein conformation.

rEhCP112 has affinity to claudin-1 and claudin-2 and it degrades and delocalizes both TJ proteins (Figures 6–8). Although the enzyme also co-localized with occludin and ZO-1, it did not produce significant visible damage on these proteins and did not associate with them. Occludin and ZO-1 proteins are involved in paracellular permeability (Liang and Weber, 2014) and their maintenance in the TJs after protease treatment is congruent with the fact that the macromolecular flux was not affected by rEhCP112a (Figure 3).

Claudin-1 restricts ions entrance to the epithelium and interacts with other claudins in the same cell and in neighbor cells, forming the fence that avoids lipid and protein diffusion (Suzuki et al., 2014), whereas, claudin-2 is present in leaky epithelia, allowing cations exit (Furuse et al., 2001), which agrees with a functional damage of TJs. Claudin-2 is higher expressed in sparse cultures, with leaky TJs, whereas in confluent monolayers, TJs are tight (Escaffit et al., 2005). When rEhCP112a disrupts TJs, the monolayers lose the cell-cell tight contact, resembling a leaky epithelium. This could explain the increase of claudin-2 in our results. Accordingly, *Salmonella* sp. also provoke an increase of claudin-2 in intestinal cell lines (Zhang et al., 2013). Interestingly, claudin-2 allows water release from the intestinal epithelium (Lu et al., 2013; Khan and Asif, 2015), which would be reflected as diarrhea symptom of amoebiasis. Additionally, Lejeune et al. (2011) also demonstrated that claudin-4 localization is altered by *E. histolytica* PGE_2_, but in our experiments the protease did not bind to or alter claudin-4, suggesting that PGE_2_ and EhCP112 have non-redundant functions (Figure 11). Here, we have analyzed only three out of more than seven claudins present in the colonic environment (Lu et al., 2013), thus, we do not exclude that rEhCP112a could affect other claudins. Intriguingly, confocal images exposed the presence of the enzyme and claudin-1 and claudin-2 in the nuclei of almost all epithelial cells in contact with it. Up to now, we do not know what is EhCP112 doing there, nor the function of claudins in the nuclei during the enzyme action.

Earlier, we found that the EhCPADH complex bound to claudin-1, occludin, ZO-1, and ZO-2 (Betanzos et al., 2013), however, rEhCP112, one of its components, only associates with claudin-1 and claudin-2 (Figure 8). Thus, it is possible that EhADH, the other component of the EhCPADH complex, could participate in the interaction with these other molecules (Figure 11).

*In vivo* EhCP112 provoked an augment in the epithelial permeability. This can be due to an increase of mucus secretion in response to the damage produced by the enzyme, which degraded claudin-1 and claudin-2. As in the *in vitro* experiments (Figure 6), EhCP112 degraded claudin-1 but not occludin (Figures 9, 10) and fluorescence strongly diminished in experiments localizing claudin-1 and E-cadherin, as reported (Hernández-Nava et al., 2017), but not in preparations stained with α-occludin antibody (Figure 10). The mice model has been previously used to study the damage produced by *E. histolytica* live trophozoites and by the recombinant EhCP5, a protease that degrades mucin (Hou et al., 2010; Kissoon-Singh et al., 2013). Mucin covers the mouse intestine and we wonder how EhCP112 reaches and damages the epithelium in spite of the presence of mucin. However, immunofluorescence staining using α-mucin-2 and α-EhCP112 antibodies revealed that EhCP112 enters through the luminal side and co-localizes with mucin in specific regions. We hypothesize that the enzyme passages across the mucin layer degrading proteins that constitute this barrier.

In conclusion, our results demonstrate that EhCP112 damages cultured epithelial cells and the intestinal epithelium, disturbing TJs through claudin-1 and claudin-2. Besides, our study reinforces the selective action of some parasite factors on certain host molecules and it also points out the concerted effect of several parasite molecules to produce the multifactorial event of virulence.

## Author contributions

PC: Designed, performed, analyzed experiments, and participated in manuscript writing. EH: Performed the *in vivo* experiments and participated in manuscript writing. GG: Participated in transfection experiments and *E. histolytica* cultures maintaining. BC: Participated in TEM experiments. MS: Participated in *in vivo* experiments. AB and EO Thesis directors of Ph.D. students PC and EH and helped to design, analyze, and discuss experiments and in manuscript writing.

### Conflict of interest statement

The authors declare that the research was conducted in the absence of any commercial or financial relationships that could be construed as a potential conflict of interest.

## Supplementary material

Figure S1 EhCP2, EhCP5, and EhCP112 alter ion flux in Caco-2 cells. **(A)** Proteolytic activity of papain, BSA and the recombinant enzymes rEhCP1, rEhCP2, rEhCP5, rEhCP112 (5 μg) was measured using the Z-Phe-Arg-AMC substrate. **(B)** Caco-2 cells were incubated with 10 μg/cm^2^ of papain or recombinant enzymes, or refolding buffer and then, TEER was measured at 5 and 90 min. TEER values were normalized according to the initial value given by each transwell (1,000 Ω/cm^2^). Means and standard errors are represented for each time point of three independent assays performed by triplicate.

Figure S2 rEhCP112a reaches the mucin layer and co-localize with mucin-2. C57/BL6 mice were treated as in Figure 9. Frozen tissue sections of were incubated with α-EhCP112 (green) and Alexa 555 labeled α-mucin2 (red) antibodies and analyzed by confocal microscopy in the *xy*-plane. White squares areas were magnified at right panels. Arrow: mucin localization, arrowheads: co-localization of mucin and EhCP112, ^*^EhCP112 localization, L: lumen. Nuclei were counterstained with DAPI.
