# Supplementary figures and images for: Entamoeba histolytica EhCP112 Dislocates and Degrades Claudin-1 and Claudin-2 at Tight Junctions of the Intestinal Epithelium
Source: Front Cell Infect Microbiol. 2017 Aug 16;7:372. doi: 10.3389/fcimb.2017.00372 (PMC5561765; doi:10.3389/fcimb.2017.00372)

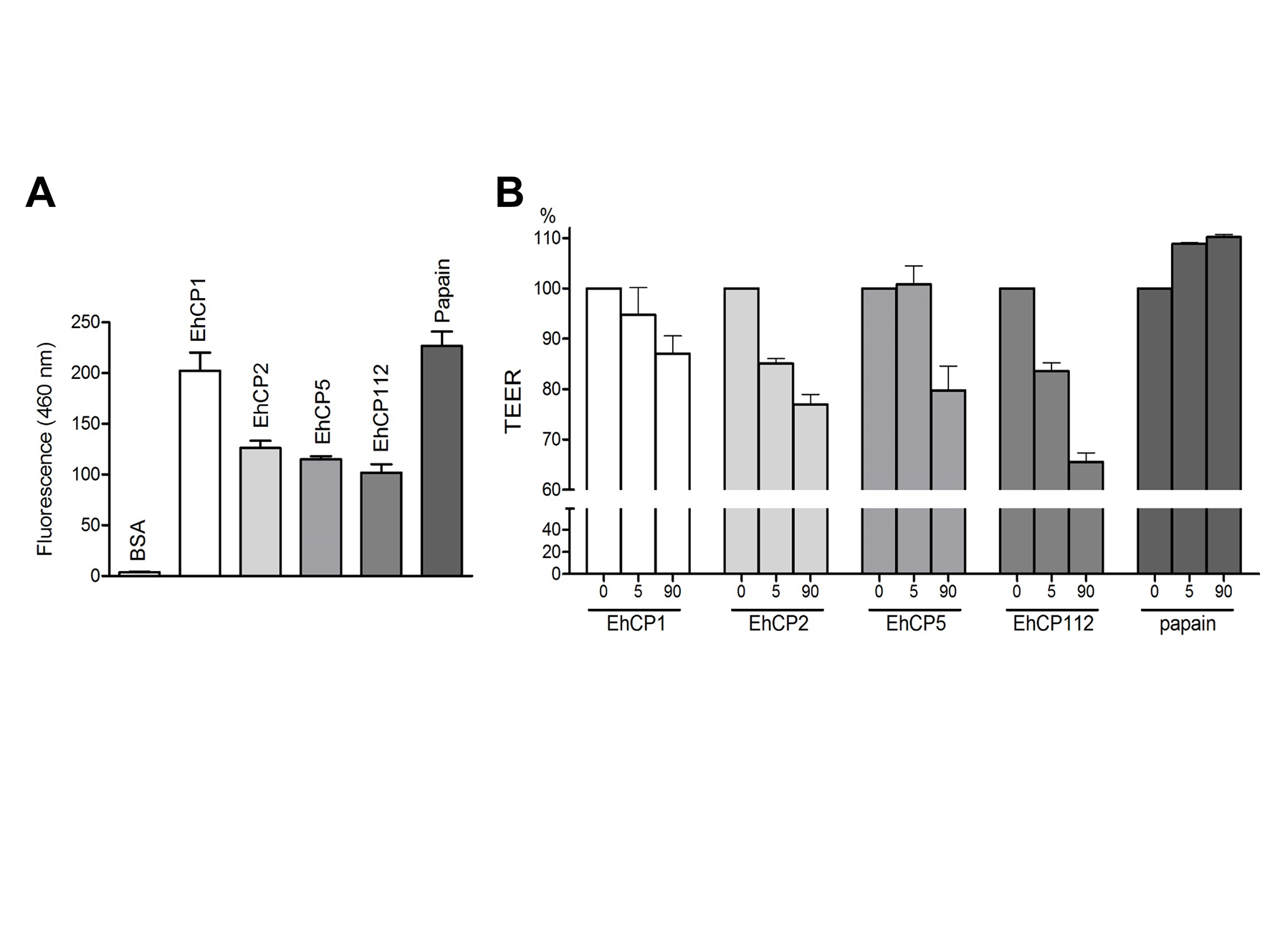

Supplement: Figure S1 — EhCP2, EhCP5, and EhCP112 alter ion flux in Caco-2 cells. (A) Proteolytic activity of papain, BSA and the recombinant enzymes rEhCP1, rEhCP2, rEhCP5, rEhCP112 (5 μg) was measured using the Z-Phe-Arg-AMC substrate. (B) Caco-2 cells were incubated with 10 μg/cm2 of papain or recombinant enzymes, or refolding buffer and then, TEER was measured at 5 and 90 min. TEER values were normalized according to the initial value given by each transwell (1,000 Ω/cm2). Means and standard errors are represented for each time point of three independent assays performed by triplicate. [file Image1.TIF]

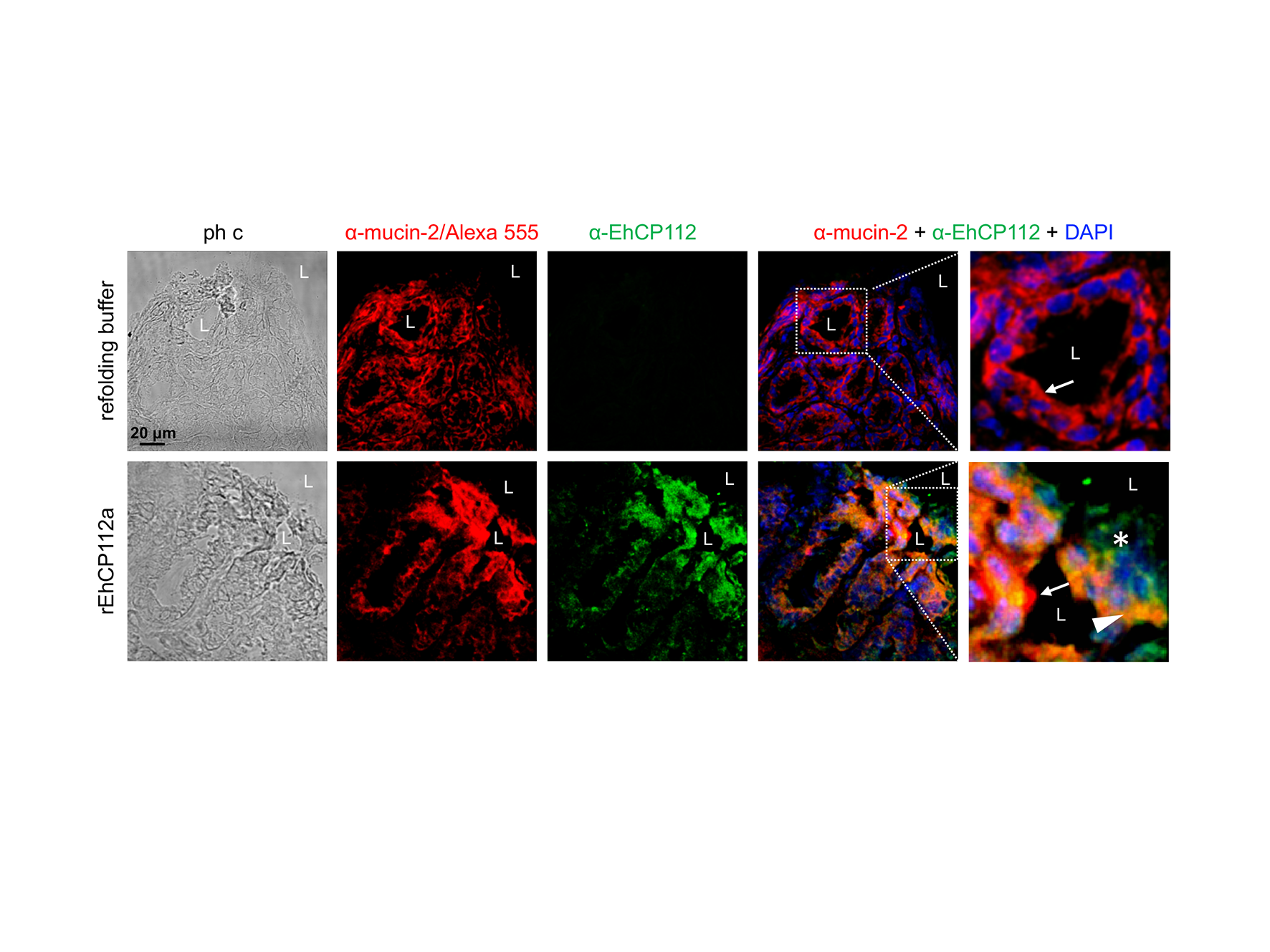

Supplement: Figure S2 — rEhCP112a reaches the mucin layer and co-localize with mucin-2. C57/BL6 mice were treated as in Figure 9. Frozen tissue sections of were incubated with α-EhCP112 (green) and Alexa 555 labeled α-mucin2 (red) antibodies and analyzed by confocal microscopy in the xy-plane. White squares areas were magnified at right panels. Arrow: mucin localization, arrowheads: co-localization of mucin and EhCP112, *EhCP112 localization, L: lumen. Nuclei were counterstained with DAPI. [file Image2.TIF]
